# Supplementary material for: The SMN complex drives structural changes in human snRNAs to enable snRNP assembly
Source: Nat Commun. 2023 Oct 18;14:6580. doi: 10.1038/s41467-023-42324-0 (PMC10584915; doi:10.1038/s41467-023-42324-0)
Supplement: Supplementary file 1 — Supplementary Information [file 41467_2023_42324_MOESM1_ESM.pdf]

Figure S1

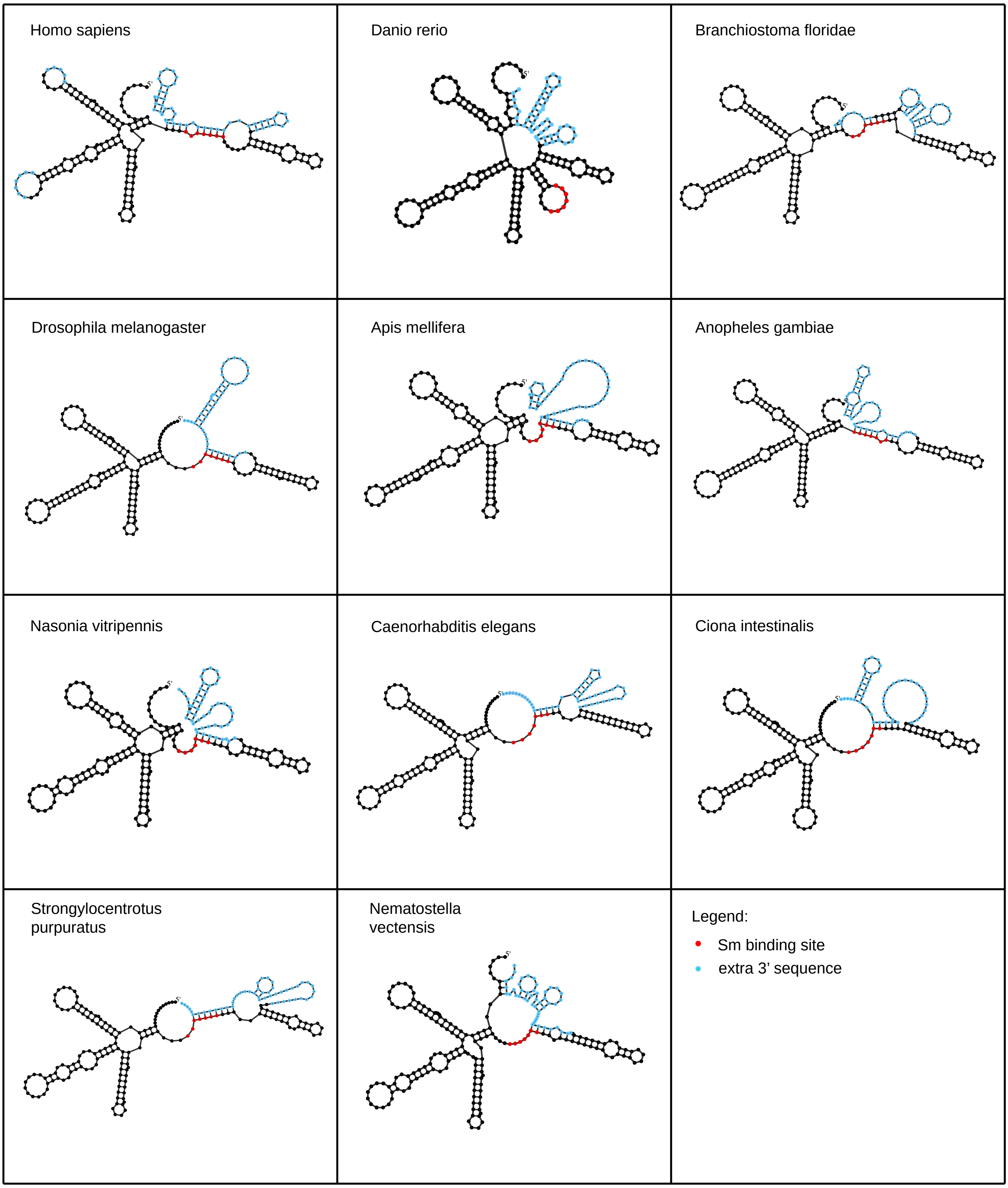

Figure S1. Structural predictions of U1 pre-snRNA primary folded transcripts.The best representative structures for 11 animal species is shown. Red circles - Sm binding site; blue circles - 3' end extension.

Figure S2

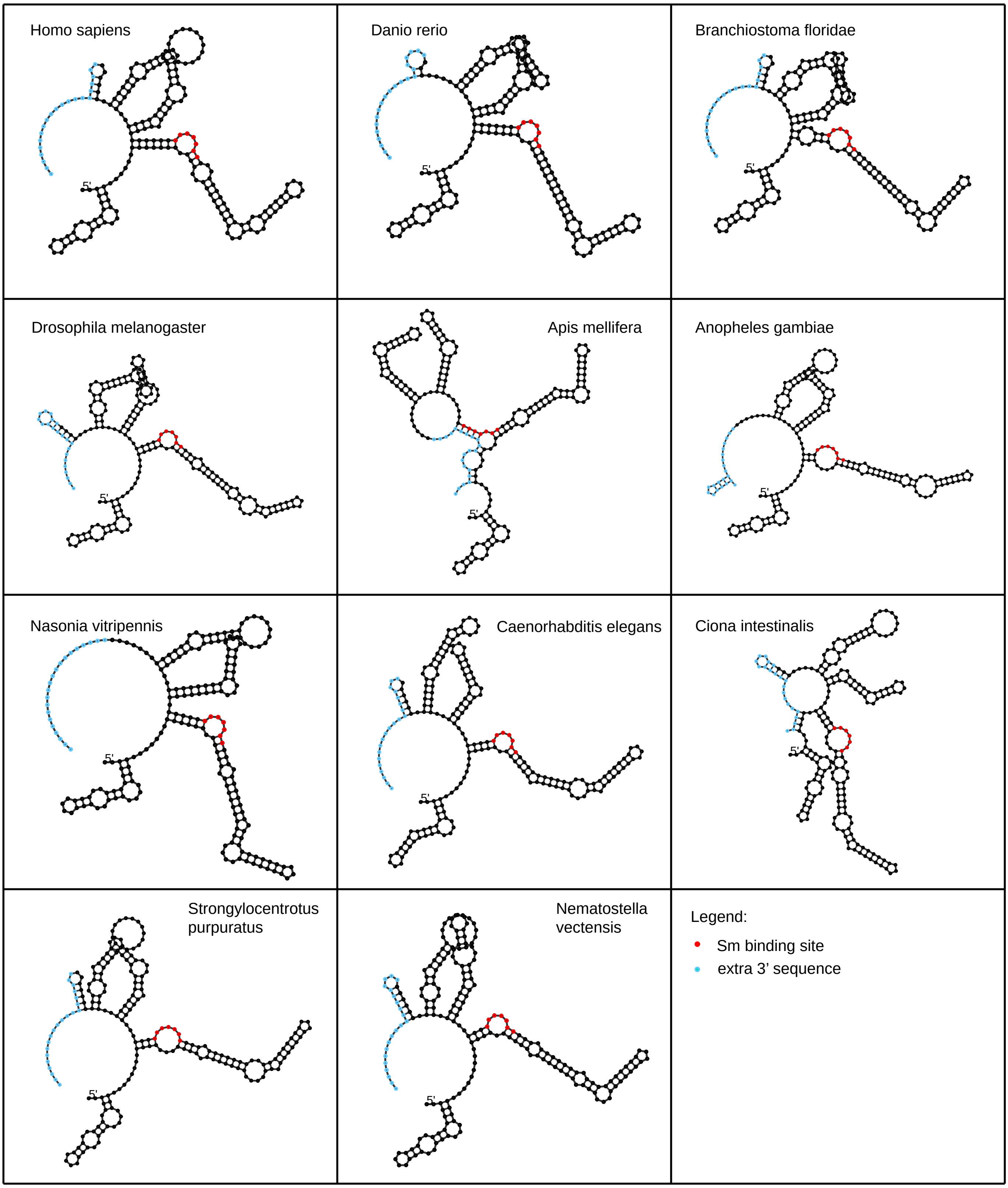

Figure S2. Structural predictions of U2 pre-snRNA primary folded transcripts. The best representative structures for 11 animal species is shown. Red circles - Sm binding site; blue circles - 3' end extension.

Figure S3

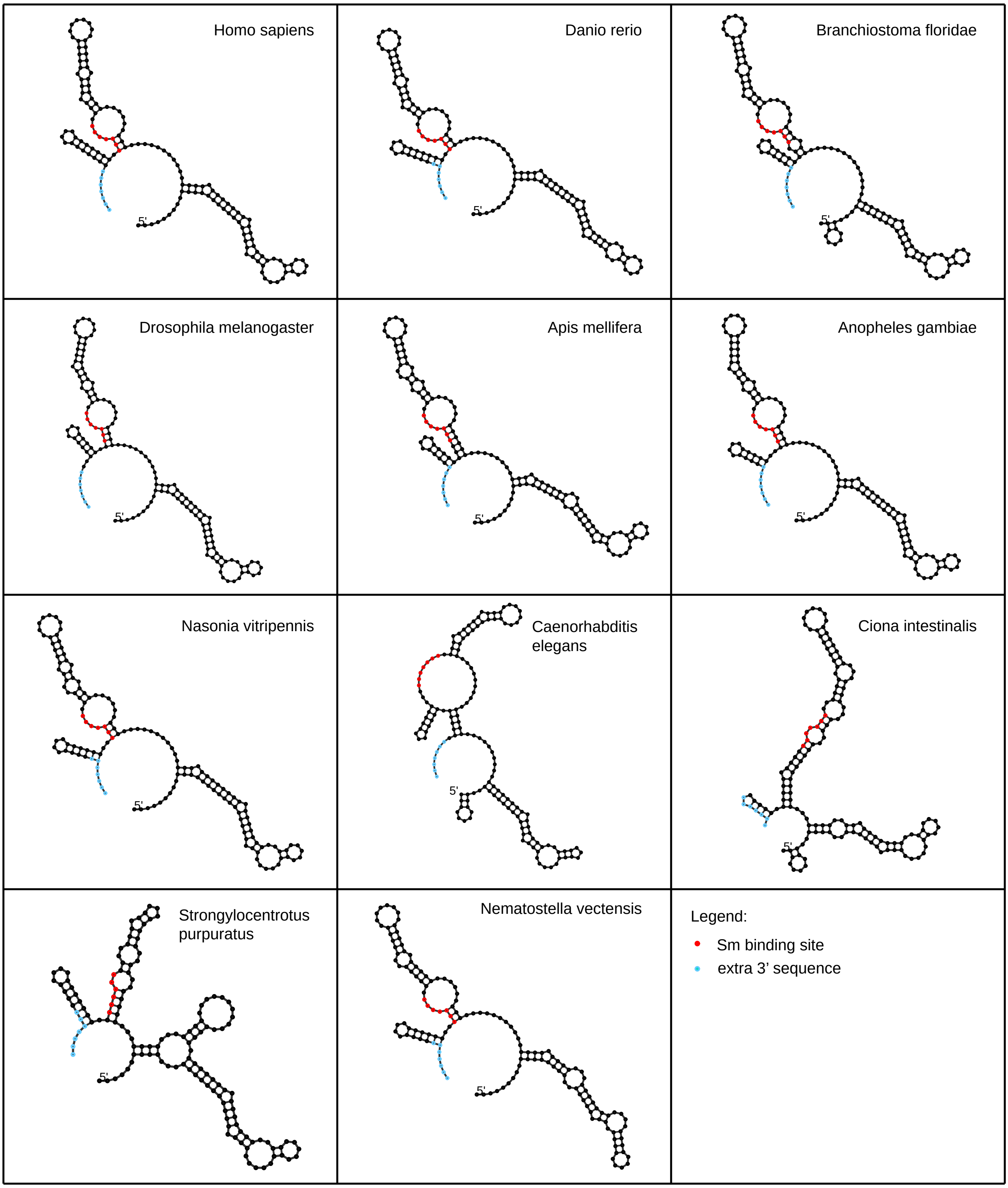

Figure S3. Structural predictions of U4 pre-snRNA primary folded transcripts. The best representative structures for 11 animal species is shown. Red circles - Sm binding site; blue circles - 3' end extension.

Figure S4

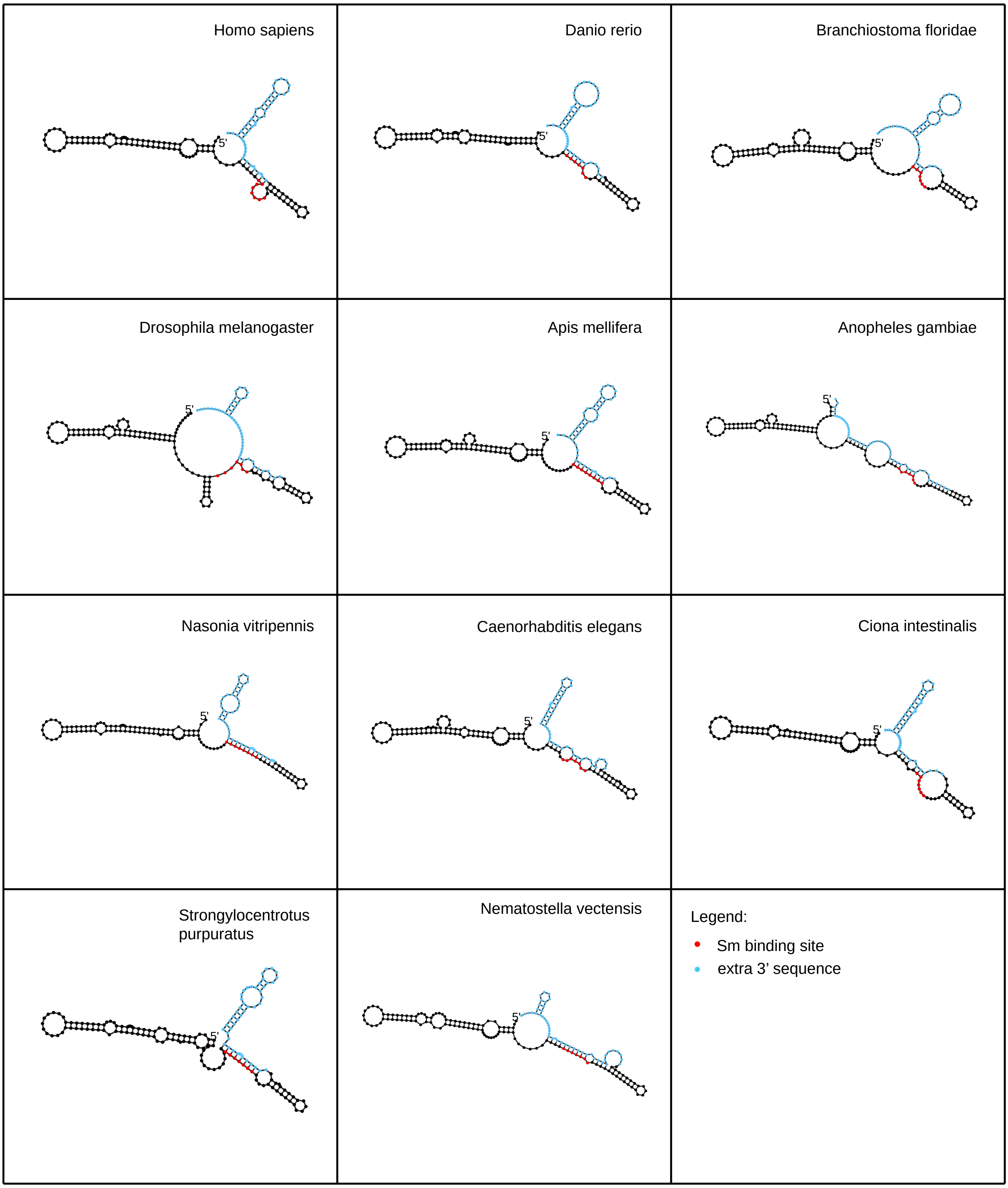

Figure S4. Structural predictions of U5 pre-snRNA primary folded transcripts.The best representative structures for 11 animal species is shown. Red circles - Sm binding site; blue circles - 3’ end extension.

Figure S5

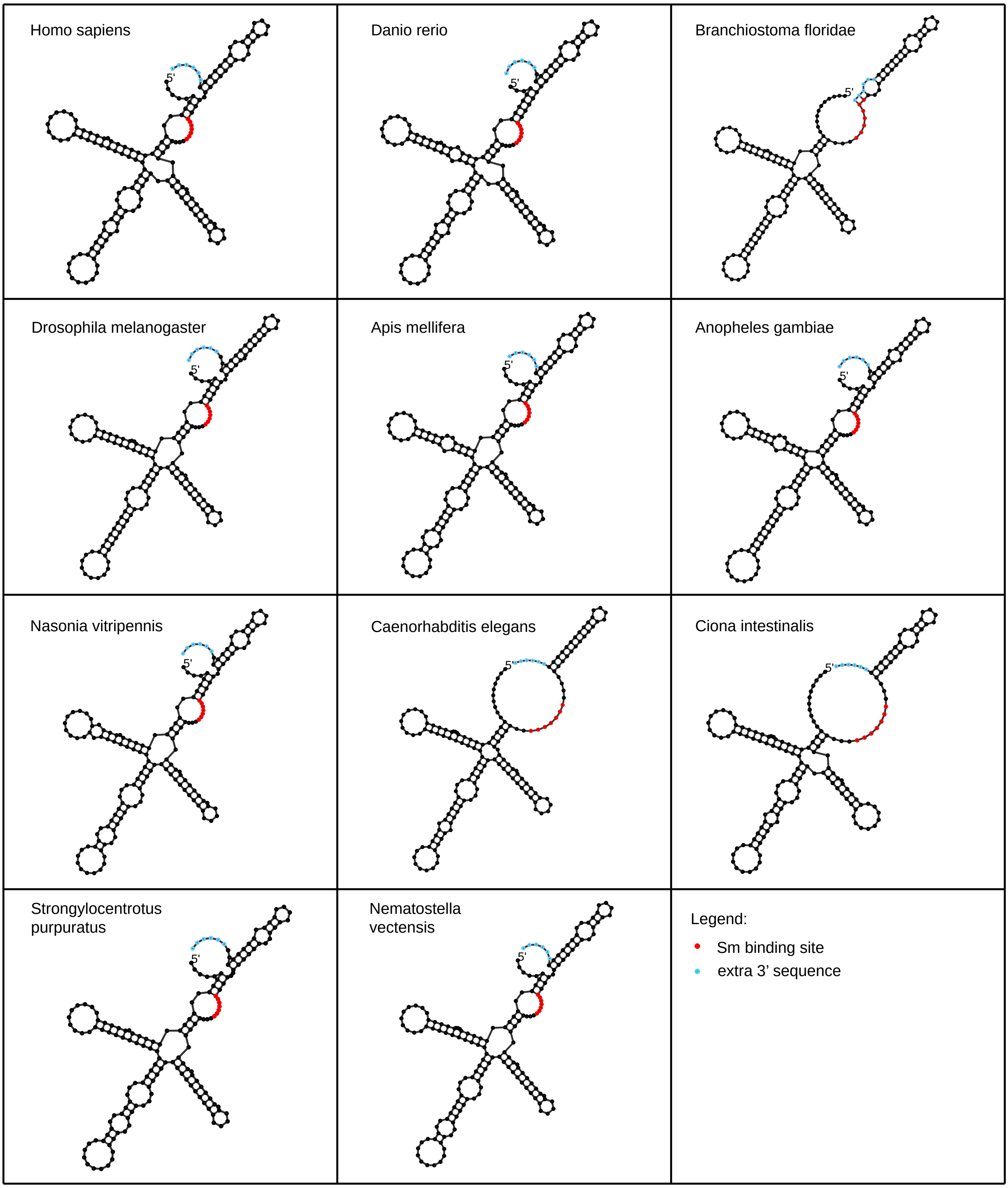

Figure S5. Structural predictions of U1 pre-snRNA primary folded transcripts with short 3' end extension. The best representative structures for 11 animal species is shown. Red circles - Sm binding site; blue circles - 3' end extension.

Figure S6

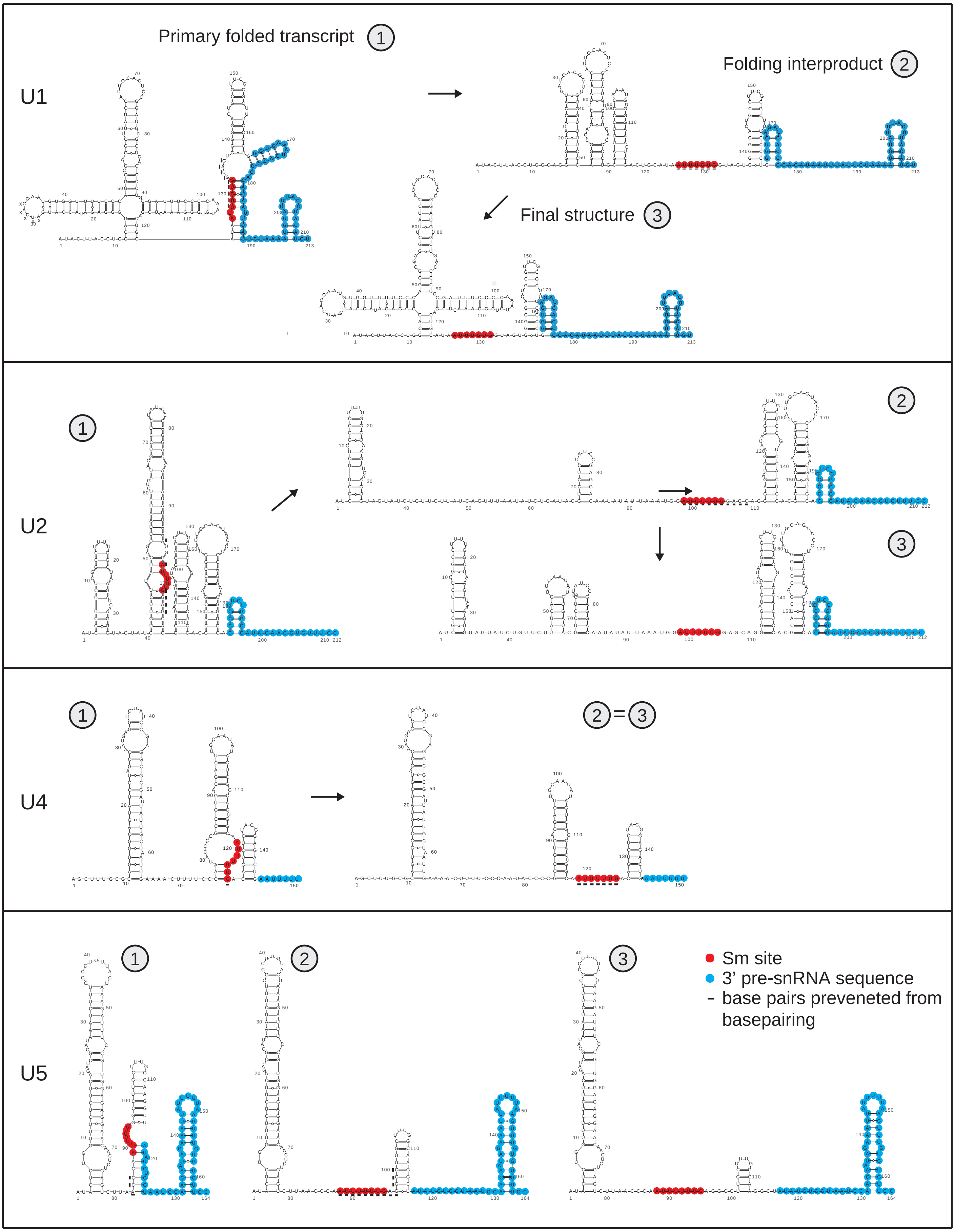

(see Figure S6 legend on the next page)

Figure S6. A detailed depiction of the predicted pre-snRNA folding pathway. It is demonstrated using secondary structures of human U1, U2, U4 and U5 pre-snRNAs with long extra sequences shown in the four panels underneath each other. The panels show each the predicted suboptimal secondary structures for primary folded transcript (①), folding interproduct (②) and final structure (③). Note that for the other evaluated species the folding pathways looked analogously as it can be seen from structures of primary folded transcripts (Figs. S1-4), folding interproducts (Figs. S7-10) and final structures (Figs. S12-15) and folding constraints shown in Supplementary data S1 and S2 in dot-bracket format. In this figure in each panel, the suboptimal secondary structure (i.e. paired and single strand nucleotides) of the primary folded transcript together with the nucleotides not allowed to pair during prediction (marked with dashes) compose the constraint for constrained prediction of secondary structure of folding interproducts. The nucleotides not allowed to pair represents a minimal constraint that model NSS opening resulting into the single stranded Sm site as described in Results section. The result of the prediction is folding interproducts. Analogously, secondary structure of folding interproducts in each panel together with new nucleotides not allowed to pair (marked with dashes) form the constraint for the prediction of final structure. In this step, the nucleotides not allowed to pair (marked by dashes) represent the minimal constraint to model steric condition caused by the presence of Sm proteins bound to the final structure which necessarily results into blocking nucleotides of both Sm site and neighboring nucleotides from pairing. Crosses in panel A are specific for U1 pre-snRNA and mark nucleotides blocked from pairing due to their interaction with U1-70K [1] protein prior folding primary transcripts. For folding constraints and secondary structures of other metazoan species, see Supplementary data S1 and S2.

Figure S7

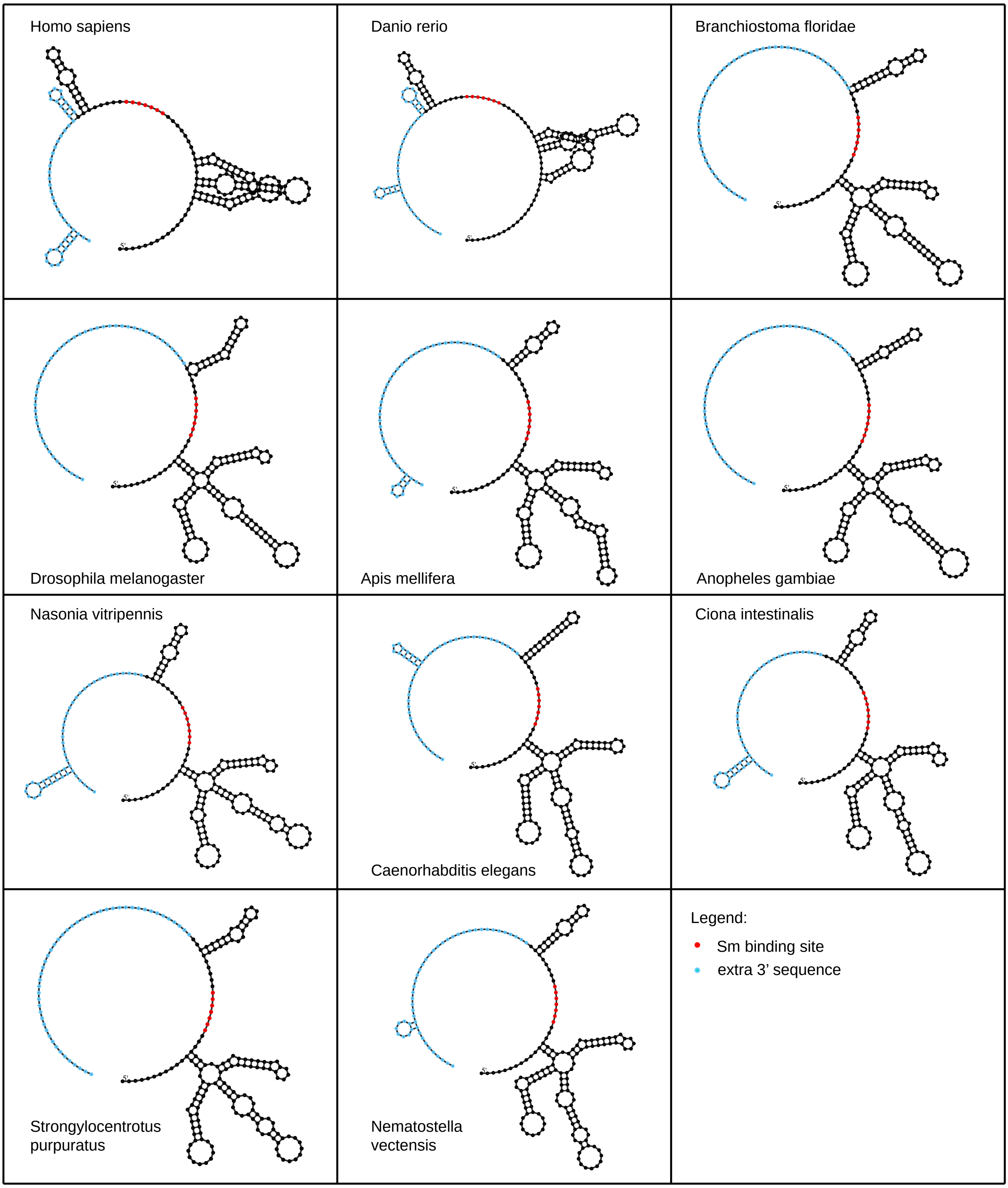

Figure S7. Structural predictions of U1 pre-snRNA folding intermediates. The best representative structures for 11 animal species is shown. Red circles - Sm binding site; blue circles - 3’ end extension.

Figure S8

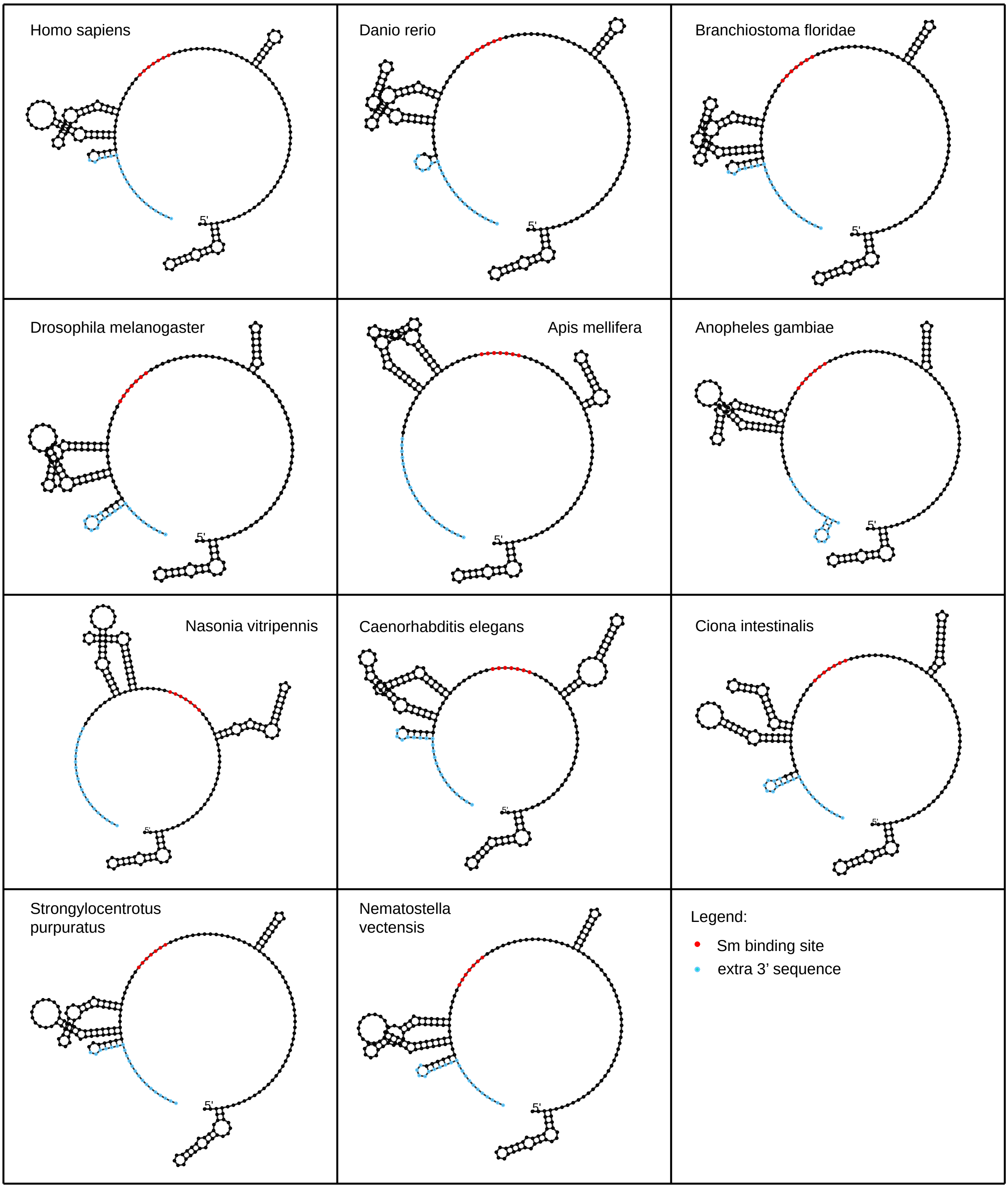

Figure S8. Structural predictions of U2 pre-snRNA folding intermediates. The best representative structures for 11 animal species is shown. Red circles - Sm binding site; blue circles - 3' end extension.

Figure S9

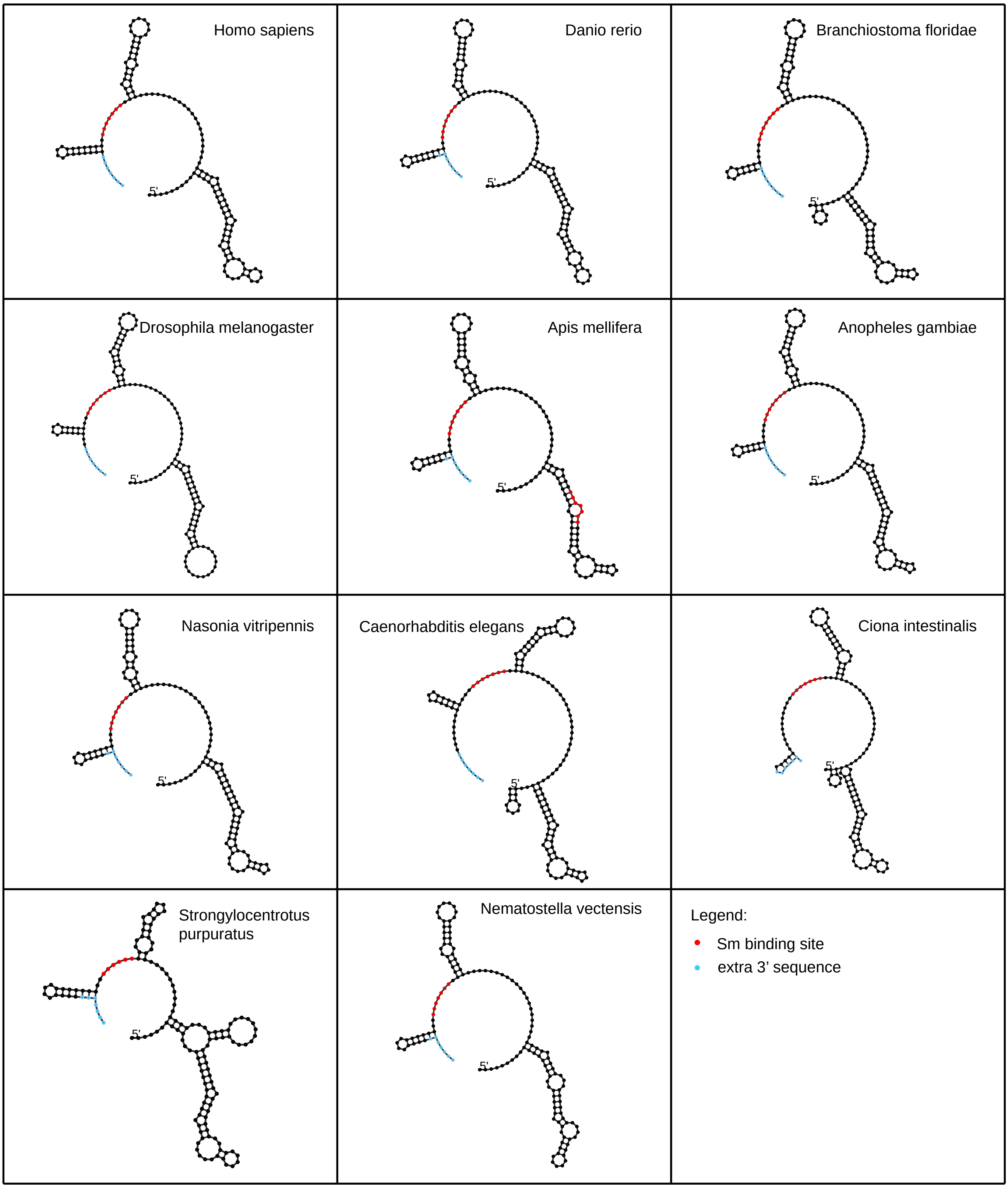

Figure S9. Structural predictions of U4 pre-snRNA folding intermediates. The best representative structures for 11 animal species is shown. Red circles - Sm binding site; blue circles - 3’ end extension.

Figure S10

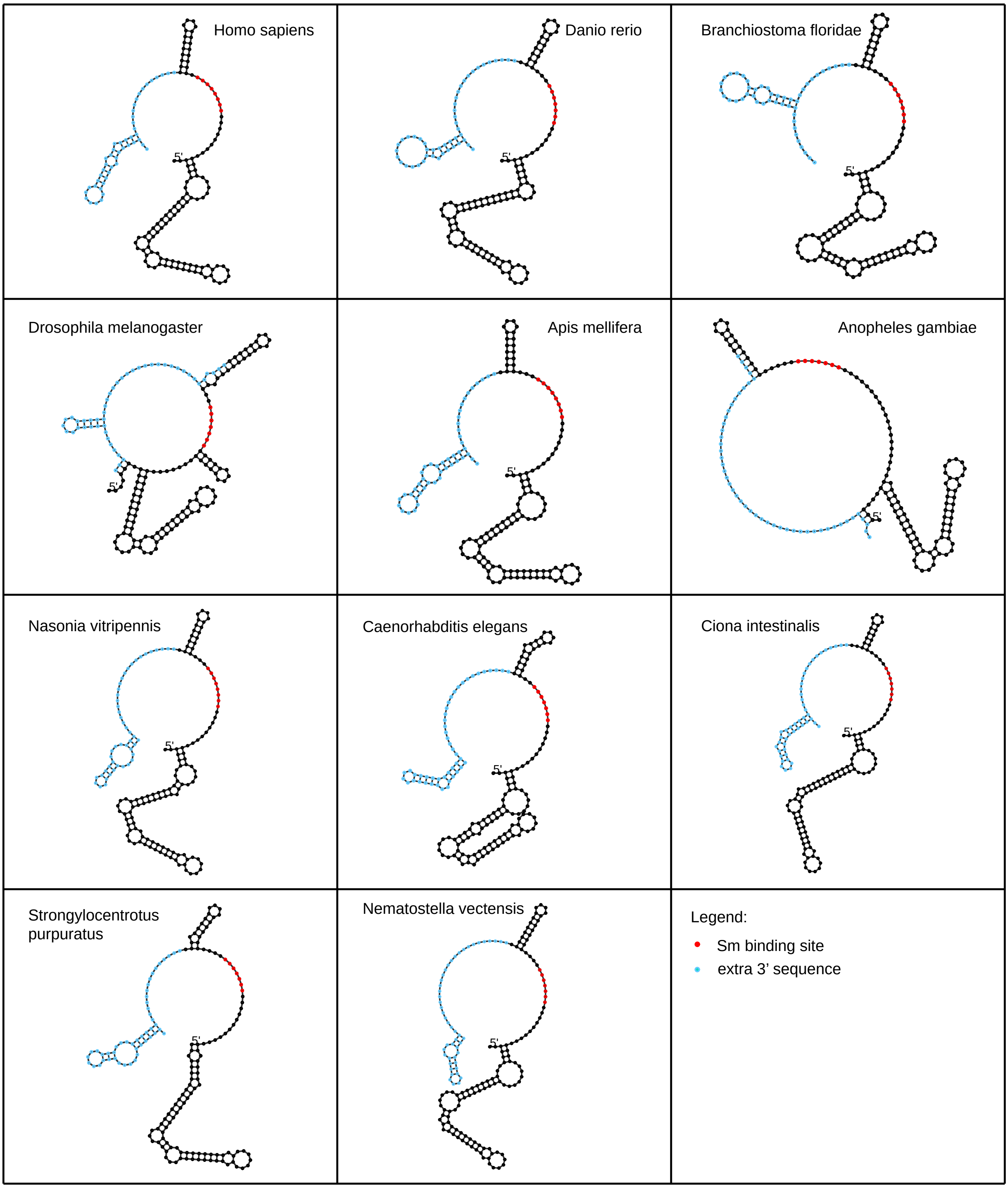

Figure S10. Structural predictions of U5 pre-snRNA folding intermediates. The best representative structures for 11 animal species is shown. Red circles - Sm binding site; blue circles - 3' end extension.

Figure S11

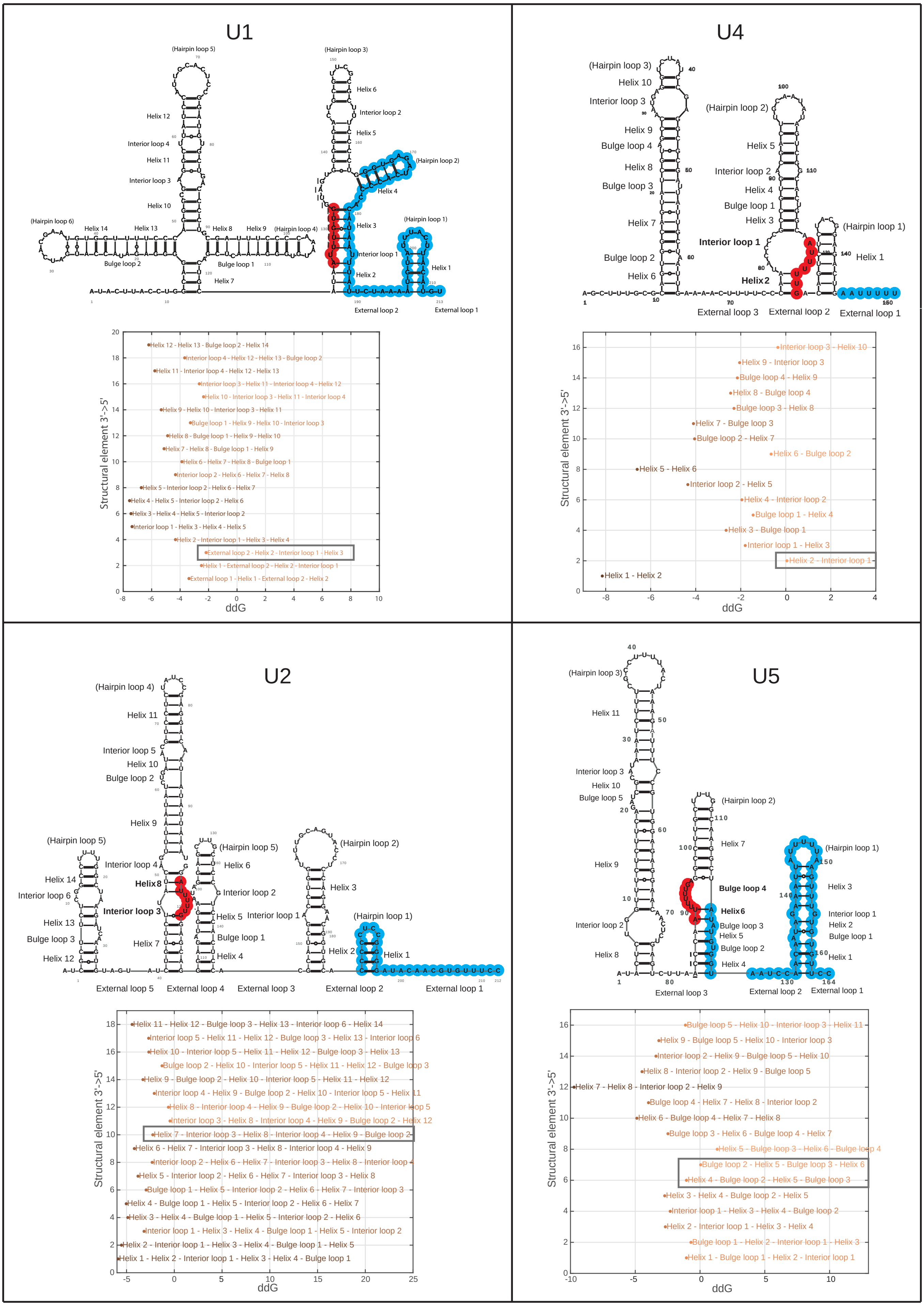

(see Figure S11 legend on the next page)

Figure S11. Thermodynamic stability of structural elements of predicted pre-snRNA structures. In the four panels, predicted secondary structures of primary folded transcripts (top) and free energy (FE) diagrams (bottom) are shown for human U1, U4, U2 and U5 pre-snRNAs, respectively. The diagrams show FEs (ddG, x-axis) of successions of structural elements (y-axis). For better readability, names of successions are colored according to their FE, the brighter color the higher FE. FEs of successions are sums of ddGs of included structure elements. The successions consist of a number of consecutive structural elements. The number of elements were chosen so for each pre-snRNA that the successions covered the restructured segments of secondary structures of primary folded transcripts. The names of successions were left-aligned to their FE values on x-axis. The figure concludes that the successions covering restructured parts of NSS are those with highest FE in structures. The elements from which the successions are assembled are shown and described in the schemes of the secondary structures. Their names consist of the type of the structure of the elements followed by their index in the structure in the 3' to 5' direction. ddGs (i.e. FEs of structure elements) were computed by Unafold as parts of FE of entire structures of primary folded transcripts, dG. dG is computed by secondary structure energy minimization. For details, see [2] and [3]. Note that the higher ddG, the more thermodynamically unstable structure element. Hairpin loops (shown in parenthesis in secondary structures schemes) were not included in the computation of FEs of structural elements successions and therefore they are not shown in the diagrams.

Figure S12

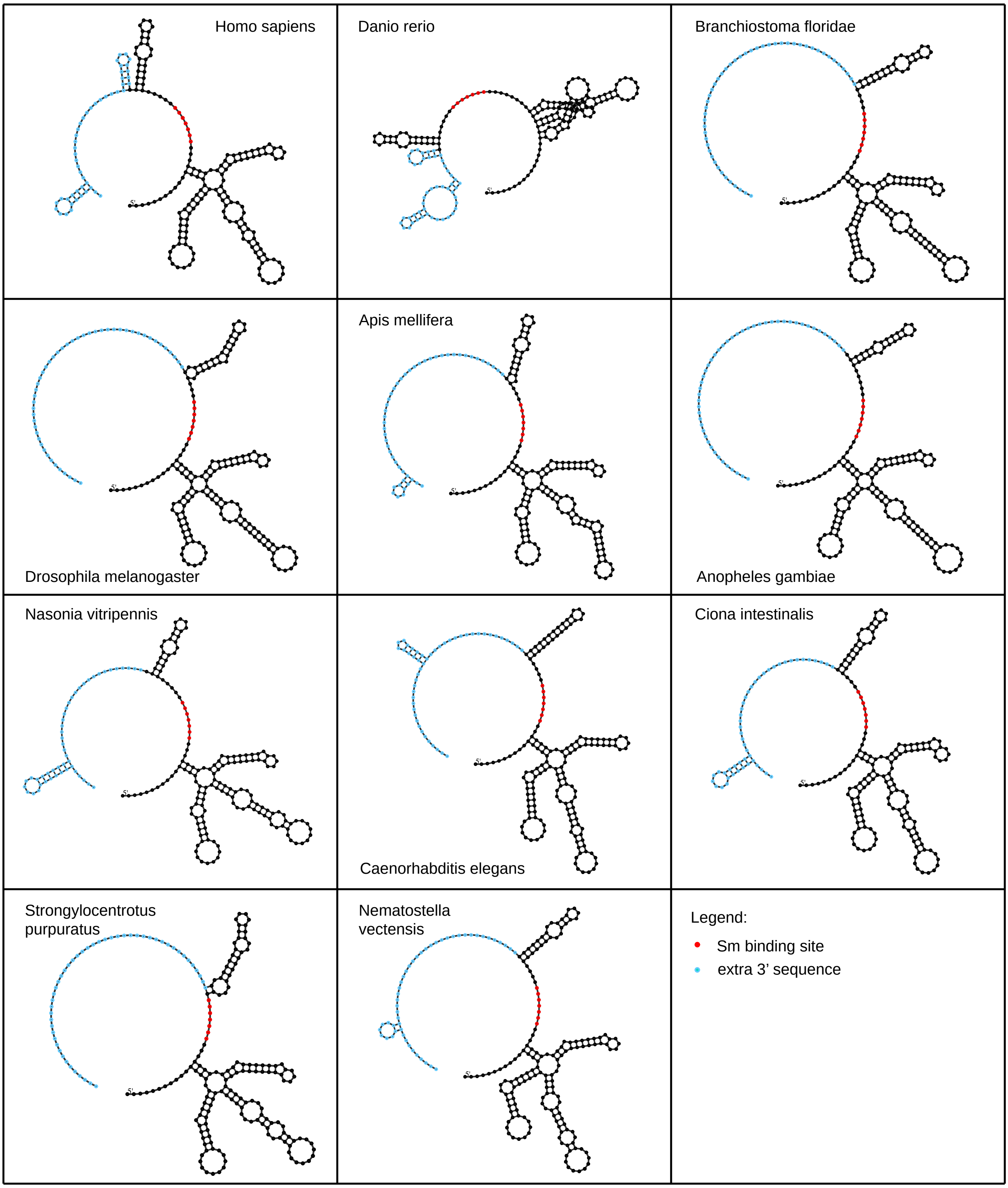

Figure S12. Structural predictions of U1 pre-snRNA final structures. The best representative structures for 11 animal species is shown. Red circles - Sm binding site; blue circles - 3’ end extension.

Figure S13

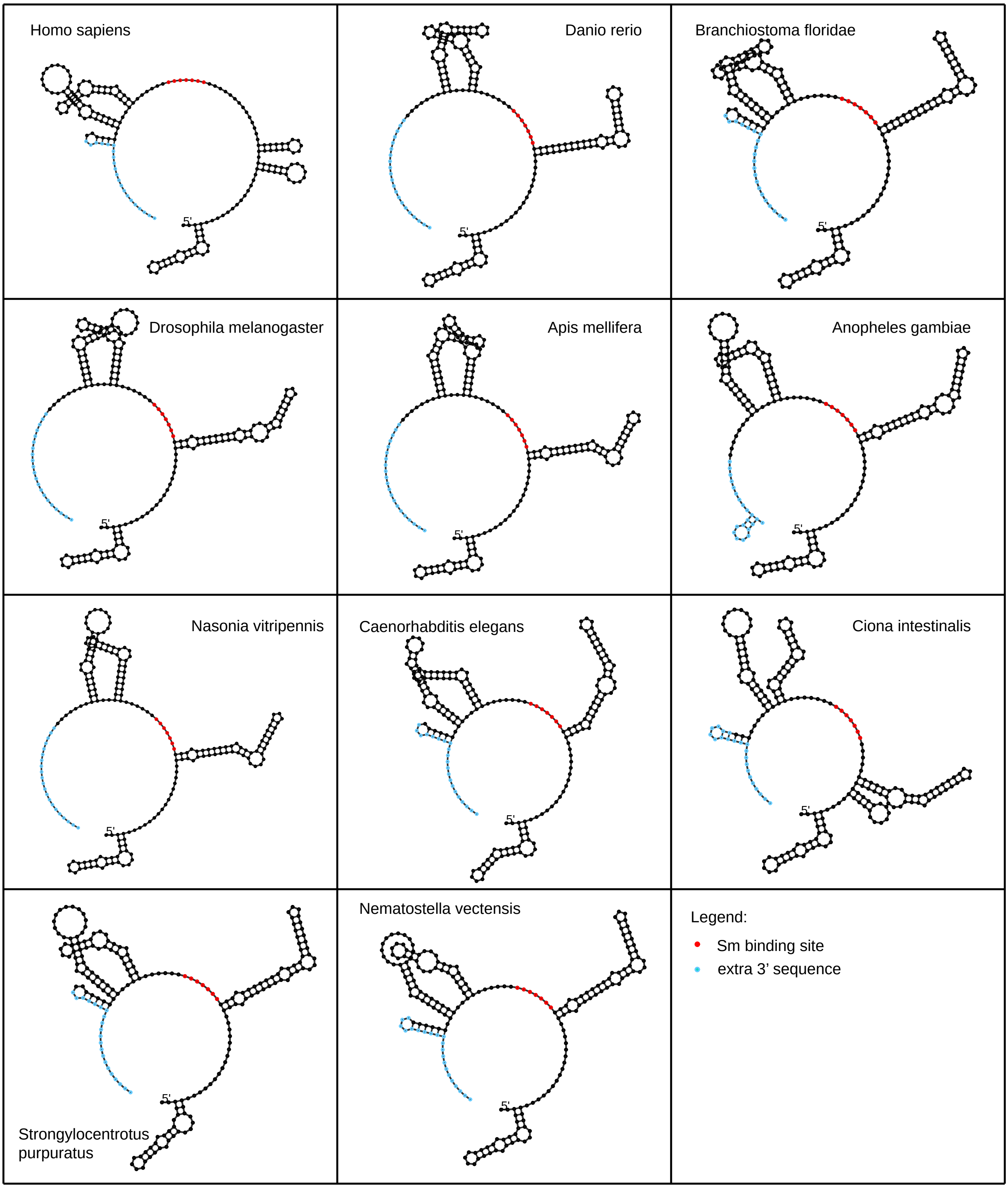

Figure S13. Structural predictions of U2 pre-snRNA final structures. The best representative structures for 11 animal species is shown. Red circles - Sm binding site; blue circles - 3' end extension.

Figure S14

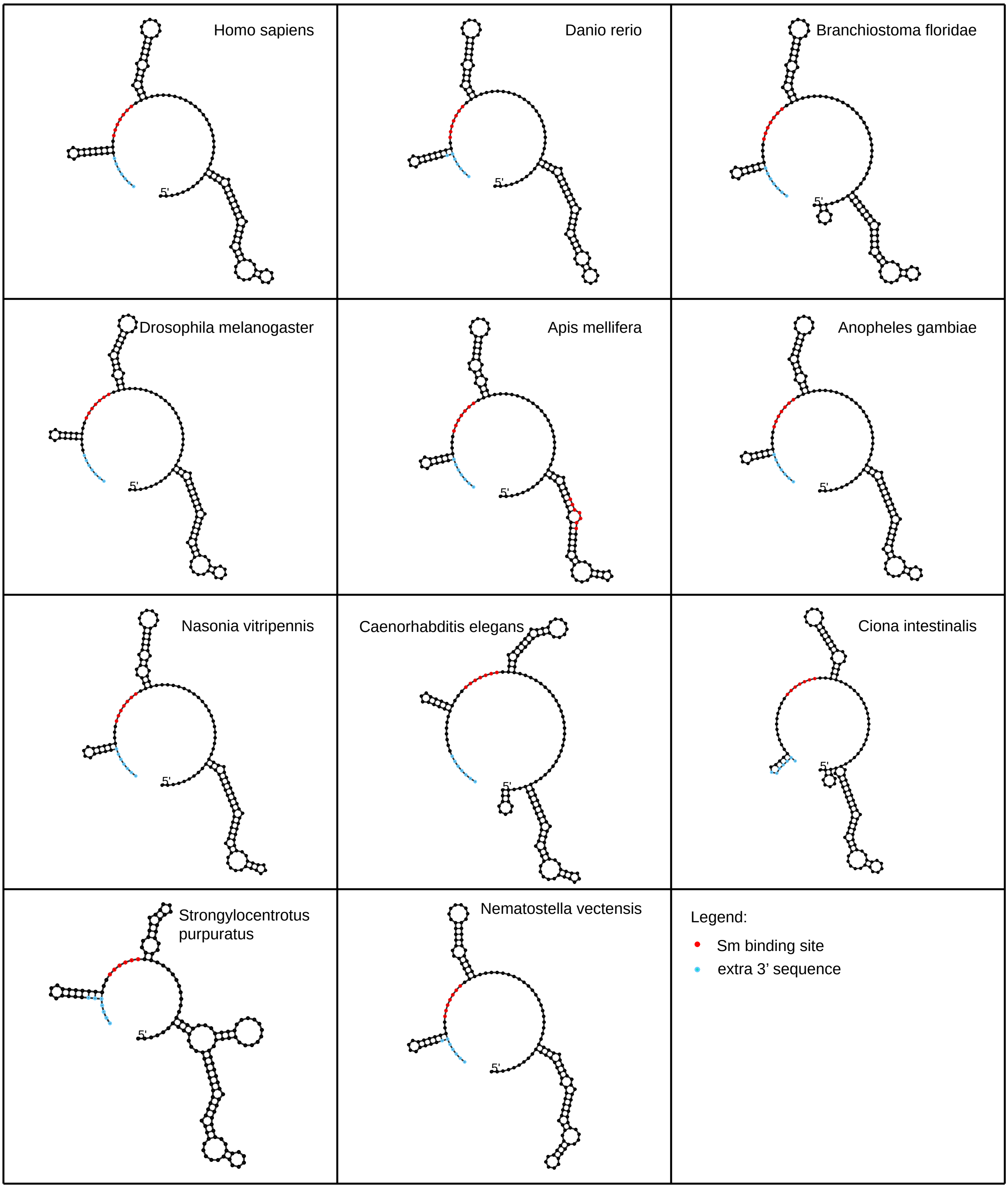

Figure S14. Structural predictions of U4 pre-snRNA final structures. The best representative structures for 11 animal species is shown. Red circles - Sm binding site; blue circles - 3' end extension.

Figure S15

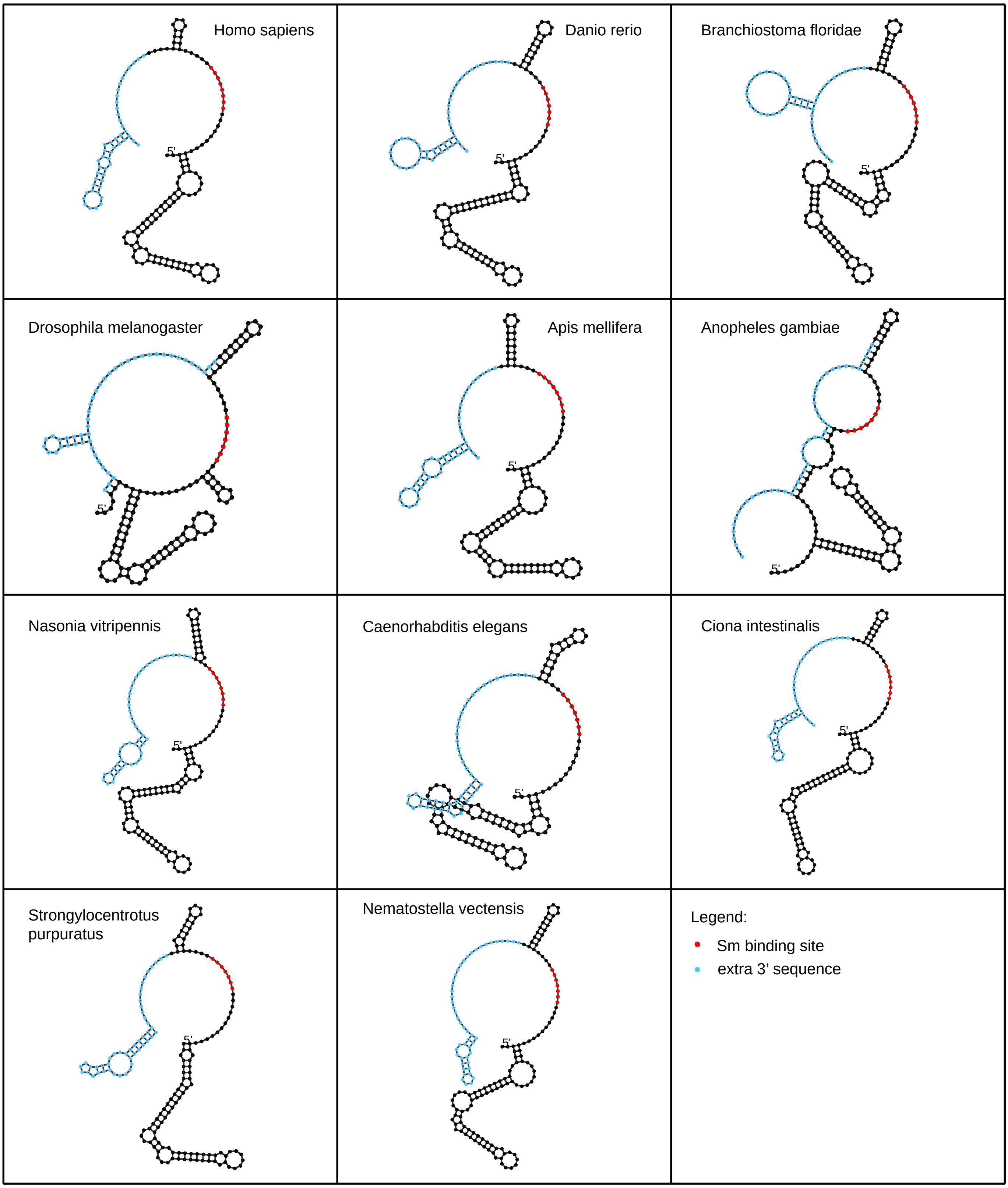

Figure S15. Structural predictions of U5 pre-snRNA final structures. The best representative structures for 11 animal species is shown. Red circles - Sm binding site; blue circles - 3’ end extension.

Figure S16

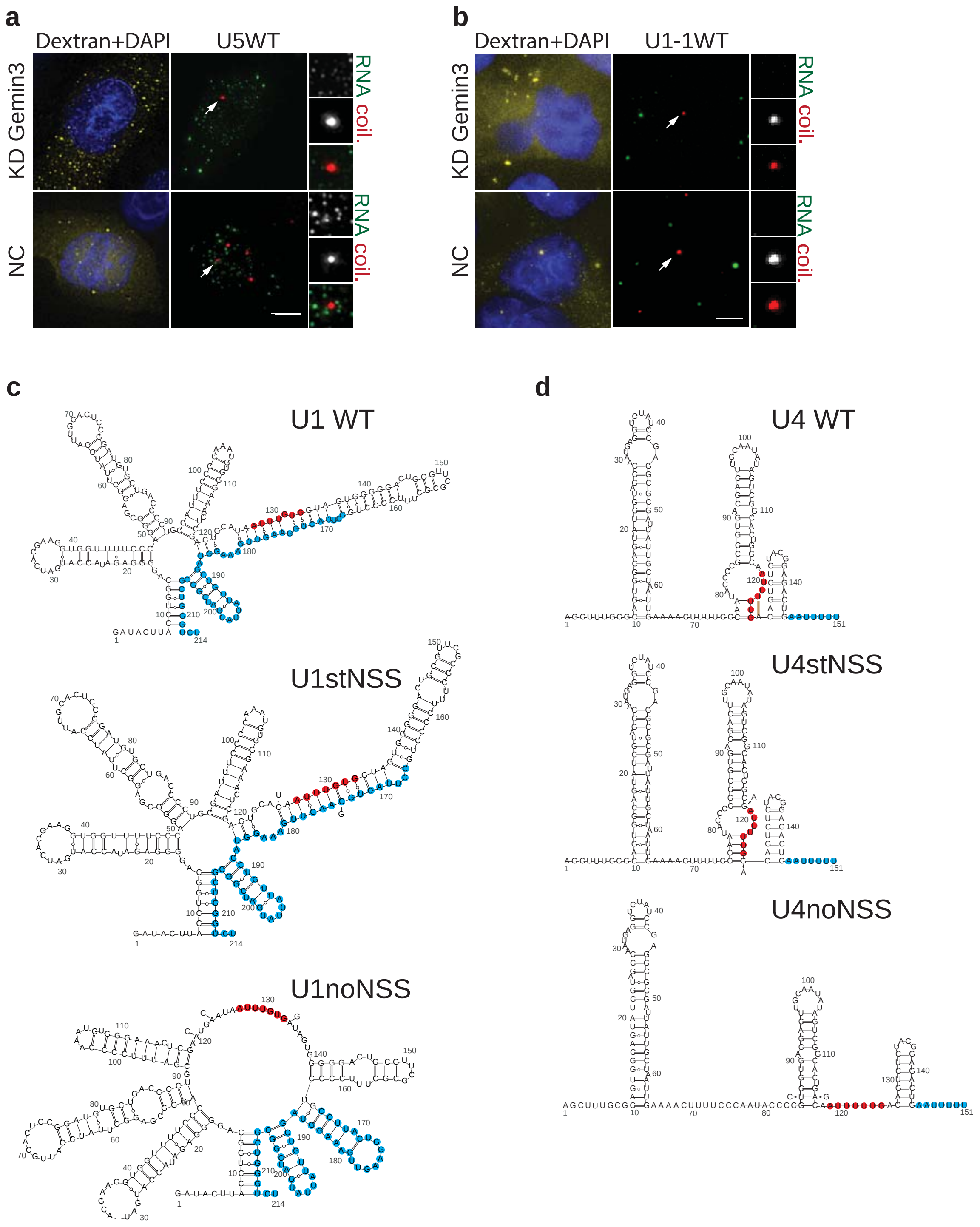

Figure S16. Microinjected U1-1 pre-snRNA and U5 pre-snRNA do not accumulate in Cajal bodies. a,b Gemin3 was downregulated by RNAi and fluorescently labeled U1-1 and U5 pre-snRNAs were microinjected into the cytoplasm. Cajal bodies marked by arrows were enlarged 3 times and shown in insets. Scale bar represents 5μm. **c, d** Predicted structure of U1-26P and U4 pre-snRNAs including noNSS and stNSS mutations. Red cicrcles - Sm binding site, blue circles - extra 3' sequence.

Figure S17

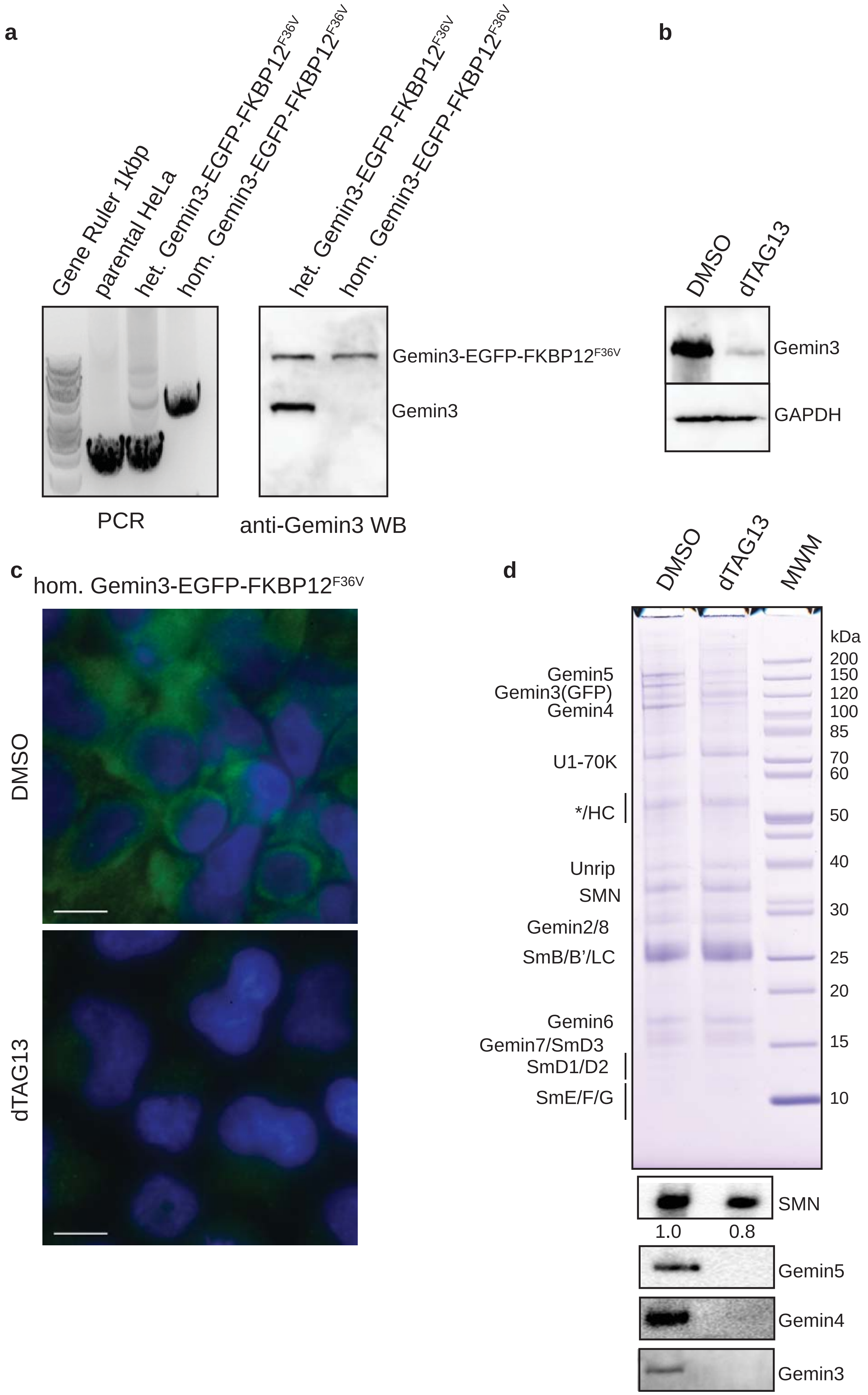
a
Gene Ruler 1kbp
parental HeLa
het. Gemin3-EGFP-FKBP12F36V
hom. Gemin3-EGFP-FKBP12F36V
het. Gemin3-EGFP-FKBP12F36V
hom. Gemin3-EGFP-FKBP12F36V
Gemin3-EGFP-FKBP12F36V
Gemin3
PCR
anti-Gemin3 WB
b
DMSO
dTAG13
Gemin3
GAPDH
c
hom. Gemin3-EGFP-FKBP12F36V
DMSO
dTAG13
d
DMSO
dTAG13
MWM
kDa
200
150
120
100
85
70
60
50
40
30
25
20
15
10
Gemin5
Gemin3(GFP)
Gemin4
U1-70K
*/HC
Unrip
SMN
Gemin2/8
SmB/B'/LC
Gemin6
Gemin7/SmD3
SmD1/D2
SmE/F/G
SMN
1.0
0.8
Gemin5
Gemin4
Gemin3

Figure S17. Characterization of Gemin3-EGFP-FKBP12^{F36V} cell line. a PCR (left panel) and western blotting (right panel) characterization of Gemin3-EGFP-FKBP12^{F36V} cell lines (homozygote and heterozygote). **b,c** Downregulation of Gemin3-EGFP-FKBP12^{F36V} upon 72h treatment of the homozygote Gemin3-EGFP-FKBP12^{F36V} cell line with 0.5μM dTAG13 assayed by western blotting (b) and fluorescent microscopy (c). GAPDH served as a loading control for western blotting. Scale bars represent 10μm in (c). **d** Analysis of the SMN complex isolated from homozygote Gemin3-EGFP-FKBP12^{F36V} cells. Cells were either mock treated (DMSO) or treated with 0.5μM dTAG13 for 72h. Proteins were resolved on SDS-PAGE gel and either stained with bromophenol blue (top panel) or western blotting (bottom panel.

Figure S18

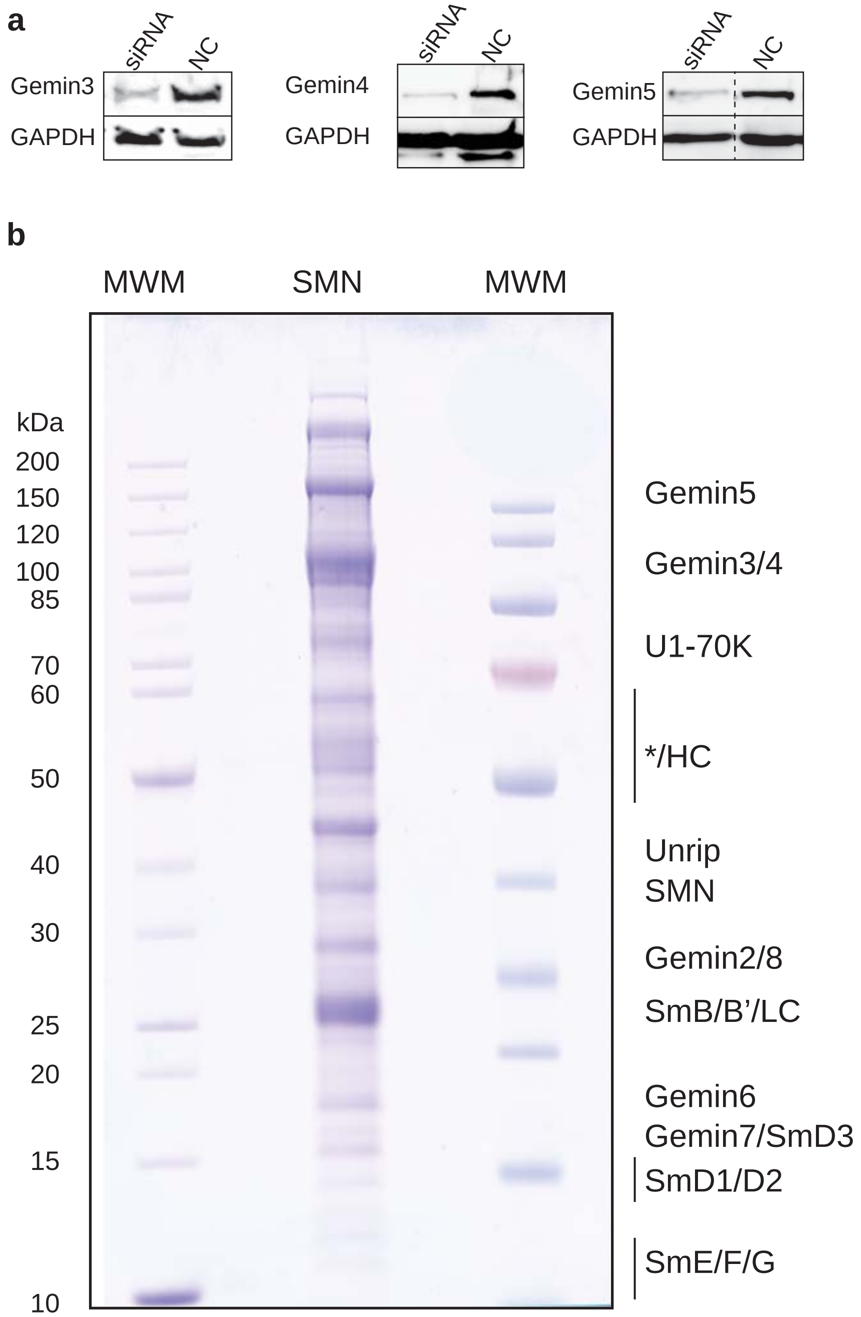

Figure S18. Knockdown efficiencies and purified SMN complex. a Downregulation of Gemin3, Gemin4 and Gemin5 was evaluated by Western blotting. Cells were treated with anti-Gemin3, anti-Gemin4 for 72h and with anti-Gemin5 siRNA for 48h. GAPDH served as a loading control. **b** SMN Complex IP from HeLa PBS cytoplasmic extract with 7B10 antibody covalently coupled to ProteinG Sepharose. Loading 25μl beads.

Supplementary Table S1

Species used in computational analysis of evolutionary structural conservation. Species were chosen to be as phylogenetically diverse as possible depending on snRNAs sequence availability using NCBI Taxonomy [4]. Numbers of input sequences and suboptimal structures predicted for them for each species are separated by slash. For each snRNA, repeating sequences, fragments (sequences shorter than 75% of average length of all sequences) and sequences without Sm motif were computationally filtered out. In case of U1 pre-snRNA, we also removed sequences lacking the U1-70K binding motif. Suboptimal structures for the pre-snRNAs were predicted by UNAfold, except for U1 pre-snRNA for which we applied RNAsubopt due to implementation of SNRNP70 binding constraint.

**Species**	**pre-U1**	**pre-U2**	**pre-U4**	**pre-U5**	**pre-U11**	**pre-U12**	**pre-U4atac**
Homo sapiens	8 / 160	23 / 460	32 / 640	19 / 380	5 / 100	2 / 40	16 / 320
Danio rerio	73 / 1251	8 / 160	13 / 260	20 / 400	2 / 40	1 / 20	1 / 20
Branchiostoma floridae	8 / 158	3 / 60	4 / 80	8 / 160	1 / 20	1 / 20	1 / 20
Ciona intestinalis	4 / 74	2 / 40	3 / 60	10 / 200	1 / 20	1 / 20	1 / 20
Strongylocentrotus purpuratus	8 / 111	5 / 100	7 / 140	7 / 140	3 / 60	2 / 40	3 / 60
Drosophila melanogaster	3 / 60	6 / 120	2 / 40	3 / 60	1 / 20	1 / 20	1 / 20
Anopheles gambiae	3 / 54	8 / 160	2 / 40	4 / 80	1 / 20	- / -	1 / 20
Apis mellifera	6 / 92	2 / 40	2 / 40	3 / 60	1 / 20	1 / 20	1 / 20
Nasonia vitripennis	6 / 99	3 / 60	3 / 60	5 / 100	1 / 20	2 / 40	1 / 20
Caenorhabditis elegans	12 / 140	10 / 300	4 / 80	11 / 220	1 / 20	1 / 20	1 / 20
Nematostella vectensis	6 / 99	6 / 120	51 / 1020	10 / 200	3 / 60	3 / 60	1 / 20
Coccidioides immitis *	1 / 20	3 / 60	1 / 20	2 / 40	×	×	×
Tuber melanosporum *	13 / 180	14 / 280	1 / 20	9 / 180	×	×	×
Schizosaccharomyces pombe *	1 / 20	1 / 20	1 / 20	1 / 20	×	×	×
Tilletiaria anomala *	1 / 20	1 / 20	1 / 20	1 / 20	×	×	×
Puccinia graminis *	2 / 40	5 / 100	2 / 40	1 / 20	×	×	×
Smittium culicis *	2 / 40	2 / 40	2 / 40	1 / 20	×	×	×
Rhizoclosmatium globosum *	5 / 80	13 / 260	8 / 160	6 / 120	×	×	×
Batrachochytrium dendrobatidis *	1 / 20	1 / 20	1 / 20	1 / 20	×	×	×
Allomyces macrogynus *	1 / 20	4 / 80	3 / 60	2 / 20	×	×	×
Linnemannia elongata *	1 / 20	2 / 40	2 / 40	1 / 20	×	×	×
Mucor ambiguus	2 / 40	2 / 40	2 / 40	1 / 20	×	×	×
Neurospora crassa **	4 / 80	4 / 80	2 / 40	1 / 30	×	×	×
Aspergillus niger **	3 / 60	3 / 60	2 / 20	1 / 30	×	×	×
Yarrowia lipolytica **	2 / 40	1 / 20	1 / 20	1 / 30	×	×	×
Rozella allomycis **	1 / 20	1 / 20	1 / 20	1 / 30	×	×	×
Reticulomyxa filosa †	3 / 60	8 / 160	4 / 80	3 / 60	×	×	×
Tetrahymena thermophila †	3 / 60	8 / 160	4 / 80	2 / 40	×	×	×
Toxoplasma gondii †	4 / 80	3 / 60	1 / 20	2 / 40	×	×	×
Plasmodium knowlesi †	1 / 20	1 / 20	1 / 20	1 / 20	×	×	×
Phytophthora sojae †	2 / 40	27 / 520	1 / 20	3 / 60	×	×	×

Dictyostelium purpureum †	2 / 40	1 / 20	1 / 20	1 / 20	×	×	×
Naegleria gruberi †	10 / 200	15 / 300	1 / 20	2 / 40	×	×	×
Guillardia theta †	1 / 20	1 / 20	1 / 20	1 / 20	×	×	×
Emiliania huxleyi †	2 / 40	3 / 60	2 / 40	6 / 120	×	×	×
Galdieria sulphuraria †	1 / 20	1 / 20	1 / 20	1 / 20	×	×	×
Amborella trichopoda †	15 / 260	25 / 440	10 / 180	11 / 220	×	×	×
Oxytricha trifallax ††	1 / 20	2 / 40	1 / 20	1 / 20	×	×	×
Cryptosporidium ubiquitum ††	1 / 20	1 / 20	1 / 20	1 / 20	×	×	×
Micromonas pusilla ††	1 / 20	1 / 20	1 / 20	1 / 20	×	×	×

* Fungi species were from 7 out of the total 11 main fungi phyla: Ascomycota: 2 species, Basidiomycota: 2 species, Zoopagomycota: 1 species, Chytridiomycota: 2 species, Blastocladiomycota: 1 species, Mucoromycota: 1 species, Cryptomycota: 1 species. The species distribution was optimized to get as diverse as possible phylogenetic distribution of species across phyla depending on snRNAs sequences availability [4,5].
** Fungi species used instead of C. immitis, T. melanosporum, T. anomala and M. ambiguous to get alternative species distribution that involved model organisms (species came from 6 out of the total 11 main fungi phyla and the species distribution was then: Ascomycota: 4 species, Basidiomycota: 2 species, Zoopagomycota: 1 species, Chytridiomycota: 1 species, Blastocladiomycota: 1, Mucoromycota: 2 species).
× Not proceeded as no conservation comparison to Metazoa was possible for metazoan pre-U11, pre-U12 and pre-U4atac snRNAs did not show structural conservation.
† Protista species were from all main phyla considered currently Protista: Sar - Rhizaria: 1 species, Sar - Alveolata: 2 species, Sar - Stramenopiles: 1 species, Amoebozoa: 1 species, Discoba: 1 species, Cryptophycea: 1 species, Haptista: 1 species, Rhodophyta: 1 species, Viridiplantae: 1 species.
†† Protista species used to get alternative species distribution used instead of T. thermophila, T. gondii and A. trichopoda.

Supplementary information references

1. Surowy, C. S., van Santen, V. L., Scheib-Wixted, S. M., and Spritz, R. A., *Mol Cell Biol* **9** (10), 4179 (1989).
2. Zuker, M., *Science* **244** (4900), 48 (1989).
3. Zuker, M., *Methods Mol Biol* **25**, 267 (1994).
4. Schoch, C.L., et al., NCBI Taxonomy: a comprehensive update on curation, resources and tools. *Database* (Oxford), 2020. **2020**.
5. James, T.Y., et al., *Nature*, 2006. **443**(7113): p. 818-22.
