# Supplementary material for: The SMN complex drives structural changes in human snRNAs to enable snRNP assembly
Source: Nat Commun. 2023 Oct 18;14:6580. doi: 10.1038/s41467-023-42324-0 (PMC10584915; doi:10.1038/s41467-023-42324-0)
Supplement: Supplementary file 3 — Description of Additional Supplementary Files [file 41467_2023_42324_MOESM3_ESM.pdf]

Description of Additional Supplementary Files

File Name: Supplementary Data 1
Description: the structure of primary folded transcripts leading to folding interproducts for individual pre-snRNA species through constrained secondary structure prediction. The files contain the sequences of individual pre-snRNAs in fasta format supplemented with constraints in dot bracket format. The sequences together with their constraints were input to the secondary structure prediction algorithm, which was RNAsubopt -C. Hyperlinks in the sequence headers refer either to the genomic sequences in which the presRNA sequences are located at the genomic loci given by the following genomic coordinates, or to the presRNA sequences. Note that some genomic IDs can be obsolete because the nucleotide databases were updated during the course of the prediction project. The best way to identify pre-snRNA sequences is to perform megaBLAST of the sequences listed below.

File Name: Supplementary Data 2
Description: Constraints used to model structure changes of folding interproducts leading to final structures for individual species of individual pre-snRNAs via the constrained secondary structure prediction. The files contain sequences of individual pre-snRNAs in fasta format, complemented with the constraint in dot-bracket format. The sequences together with their constraints were the input to the secondary structure prediction algorithm that was RNAsubopt –C. For hyperlinks of genomic IDs , please see Supplementary Data 1.

File Name: Supplementary Data 3
Description: A list of primers used

File Name: Supplementary Software 1
Description: The theoretical background for the provided code is explained in the Computational procedures section in Methods. Contacting the authors is strongly recommended before the use of the presented code, as the code is not a classical input –> code – > output software, but rather computational analytical tools that are used with several manual steps.

The code uses both Matlab and Linux bash programing languages. Running the Matlab scripts (*.m) requires both a Linux operating system and the Matlab computational environment with the Bioinformatics and Statistics toolboxes. The scripts contain a substantial portion of the Linux bash code. For the presented work, Matlab v. 2019a and CentOS 7 were used, nevertheless, the code is version-independent. No installation is required for the scripts. No software repository was used to provide the code as it is a single-use, single– purpose and single-version software, and there will be no future development. No demo data is provided as the analysis includes manual steps. The results of the computation using the provided scripts is not self-explanatory and should be interpreted visually.
