# Supplementary material for: The SMN complex drives structural changes in human snRNAs to enable snRNP assembly
Source: Nat Commun. 2023 Oct 18;14:6580. doi: 10.1038/s41467-023-42324-0 (PMC10584915; doi:10.1038/s41467-023-42324-0)
Supplement: Supplementary file 4 — Supplementary Data 1 [file 41467_2023_42324_MOESM4_ESM.zip › Supplementary Data-1.docx]

**Supplementary Data 1**

Constraints used to model changes in the structure of primary folded transcripts leading to folding interproducts for individual pre-snRNA species through constrained secondary structure prediction. The files contain the sequences of individual pre-snRNAs in fasta format supplemented with constraints in dot bracket format. The sequences together with their constraints were input to the secondary structure prediction algorithm, which was RNAsubopt -C. Hyperlinks in the sequence headers refer either to the genomic sequences in which the pre-sRNA sequences are located at the genomic loci given by the following genomic coordinates, or to the pre-sRNA sequences. Note that some genomic IDs can be obsolete because the nucleotide databases were updated during the course of the prediction project. The best way to identify pre-snRNA sequences is to perform megaBLAST of the sequences listed below.

**U1**

>13_u1_28_1_pre2_[XR_0 XR_007067098](https://www.ncbi.nlm.nih.gov/nuccore/XR_007067098.1)

[07067098.1](https://www.ncbi.nlm.nih.gov/nuccore/XR_007067098.1)_PREDICTED_Homo_sapiens_U1_spliceosomal_RNA_LOC124904616_ncRNA

AUACUUACCUGGCAGGGGAGAUACCAUGAUCACGAAUGUGGUUUUCCCAGGGCGAGGCUUAUCCAUUGCACUCCGGAUGUGCUGACCCCUGCGAUUUCCCCAAAUGUGGGAAACUCGACUGCAUAAUUUGUGGUAGUGGGGGACUGCGUUCGCGCUUUCCCCUGGGUGAGAUCACCCCACAUAAUUUAUUCUAAAAUGUAUUUACUUACAUGU

...........xx((((((((.((((((........))))))))))))((((.(.(((.(((((..........))))).)))..))))).((((((((((....).)))))).))).))..((((.xxxxxxxxx.((((((..(((....)))..))))))(((((....)))))........))))......(((((......)))))..

>9_u1_pre2_[CM002888.2](https://www.ebi.ac.uk/ena/browser/view/CM002888)_69962711-69962548_Danio_rerio_chromosome_4_GRCz11_reference_primary_assembly.

AUACUUACCUGGCAGGGGAGAUACCAUGAUCAAGAAGGUGGUUCACCCAGGGCGAGGCUUGGCCAUUGCACUCCGGCCACGCUGACCCCUGCGAAUUCCCCAAAUGUGGGAAUCUCGACUGCAUAAUUUCUGGUAGUGGGGGACUGCGUUUGCGCUCUCCCCUGAGCACAGUGGUUCAAGACAGAGUUCAGGCGGUAGCGUCGGACCCGGCUG

..........xxx.((x(.((.(((((..........))))))).))(((((.(.(((.(((((..........))))).)))..))))))((((((((((....).)))))).)))............xxxxxxxx((((((..(((....)))..))))))((((......))))........((((.((((....))))))).))xxxxx

>14_u1_pre2_[GG666660.1](https://www.ncbi.nlm.nih.gov/nuccore/GG666660.1)_4494-4657_Branchiostoma_floridae_genomic_scaffold_BRAFLscaffold_244_whole_genome_shotgun_sequence.

AUACUUACCUGGCAGAGAUGGCACAUUGAUCACGAAGGAUGUCCGUCCAGGGCGAGGCCCGACCAUUGCACUCCGGUUGGGUUGACCCUUGCGAUUACCCCAAAUGCGGGUAACUCGGCUGCGUAAUUUAUGAUAGUGGGGGUCUGCGUUCGCGCUAACCCCCGCCUUUACAAUCCAAAAGCAAGUAUACAACACACCUGAUCAUAUCCGACA

...........((((.(((((.(((((..........))))))))))(((((.(.(((((((((..........)))))))))..))))))((((((((((....).)))))).))).))))((....xxxxxxx((((((((..(((....)))..))))))))((((........))))........((.......))..........)).

>6_u1_pre2_[AABS01000073.1](https://www.ncbi.nlm.nih.gov/nuccore/AABS01000073.1)_3124-2961_Ciona_intestinalis_ciona0073_whole_genome_shotgun_sequence.

AUACUUACCUGACGAGGGCCUUCACUGCGAUCAGGCAGGCAGUGGGUCCAAGGUGAGGCCAGGCCAUUGCACAUCGGCUUAGCUGACCCUUGCGAUUACCCUAAAUGUUGGUAACUCGGUCGUAUAAUUUCUGCUAGUGGGGACUGCGUUCGCGCUAUCCCCGCCCCAAUCUUUAAUCCUAGUUUAGCAAAACAAGUACAUGAACUUGUGUAC

...........(((((((((..((((((..........)))))))))))((((.(.(((.(((((..........))))).)))..))))).((((((((.........))))).))).))))........xxxxxxx(((((..(((....)))..))))).............................((((((......))))))....

>7_u1_pre2_[AAGJ05083895.1](https://www.ncbi.nlm.nih.gov/nuccore/AAGJ05083895.1)_7779-7940_Strongylocentrotus_purpuratus_Contig83895_fixed_whole_genome_shotgun_sequence.

AUACUUACCUGGCGCAGGGGUCGCAUUGAUCAAGAAGGAUGCACCCCCAGGGCGAGGCUUGCCAUUGCACUCCGGCUUGCUGAAUCUUGCGAUUCCCCCAAACGUGGGGAACUCGGGCGUAUAAUUUAUGGUAGCGGAGAUCUGCGUUCGCGCUAUCUCCUACAAUUCAAAAUGCAAAGAAAGAAAAACAUCGAAAAAUAUCAUGACAUUU

...........((((.(((((.(((((..........))))))))))(((((...(((..(((..........)))..)))...)))))((((((((((....).)))))).))).)))).....xxxxxxxx..((((((..(((....))).))))))......................((......))...................

>13_u1_pre2_[AE014297.3](https://www.ncbi.nlm.nih.gov/nuccore/AE014297.3)_23826334-23826498_Drosophila_melanogaster_chromosome_3R.

AUACUUACCUGGCGUAGAGGUUAACCGUGAUCACGAAGGCGGUUCCUCCGGAGUGAGGCUUGGCCAUUGCACCUCGGCUGAGUUGACCUCUGCGAUUAUUCCUAAUGUGAAUAACUCGUGCGCGUAAUUUUUGGUAGCCGGGAAUGGCGUUCGCGCCGUCCCGACAUUUAAAAUAAAUAUUGAAAAGAAAACGACUCAACUUUAGUUAAUAAUU

...........(((((((((..((((((..........))))))))))(((((.(.(((((((((..........)))))))))..))))))((((((((((....).)))))).)))))))).....xxxxxxxx..(((((.(((((....))))))))))..............((((((((((............))))..))))))...

>14_u1_pre2_[EQ090204.1](https://www.ncbi.nlm.nih.gov/nuccore/EQ090204.1)_7076076-7075911_Anopheles_gambiae_M_scf_1925491376_genomic_scaffold_whole_genome_shotgun_sequence.

AUACUUACCUGGCACAGGGGUUACCGUGAUCACGAAGGCGGUUCCCCCAGGGCGAGGCCUGGCCAUUGCACACUUAGGCUGGGUUGACCCCUGCGAUUAUCCCUCAUGUGGAUAACUCGUGUGCGCAAUUUUUGGUAGCCGGGAAGUGCGUCCGCGCACAUCCCGAUUUAAUCUGAAAUACAAAACAAAUAUAUGUCAAGUUUUAACUUUUUACA

.........(((((((((((..(((((..........))))).))))(((((.(.(((((((((............)))))))))..))))))((((((((((....).)))))).)))))))).))(((xxxxxxxxx(((((.(((((....))))).)))))...........)))..............(((.((((....))))...)))

>6_u1_pre2_[CM000069.5](https://www.ncbi.nlm.nih.gov/nuccore/CM000069.5)_7012443-7012279_Apis_mellifera_strain_DH4_linkage_group_16_whole_genome_shotgun_sequence.

AUACUUACCUGACGCAGAGGUUACCGUGAUCAUAAAGGCGGUUCCUCCAGGGCGAGACUCGUCCAUUGCACUUGUGGAUCUGAGCUGACCCCUGCGAAUGCCCCUAAUGCGGGUGUCUCGGGCGUAAAAUUUUUGGUAGUCGGGACUGCGUUCGCGCUAUCCCGCGAAUAUUAAUUAAAAAAAUAAUAAUAAUAUAACAUUCUCCAAUUGGAAA

...........((((.((((..(((((..........))))).))))(((((..((.(((((((((.......))))))..)))))...)))))((((((((((....).)))))).))).)))).......xxxxxxxx(((((..(((....)))..)))))......................................(((....)))..

>19_u1_pre2_[CM000916.2](https://www.ncbi.nlm.nih.gov/nuccore/CM000916.2)_26139276-26139115_Nasonia_vitripennis_chromosome_2_whole_genome_shotgun_sequence.

AUACUUACCUGGCGCAGAGGCUACCGUGAUCACGAAGGCGGUUCCUCCAGGGCGAGGCUCUUCCAUUGCACUACGGUCGAGCUGACCCUUGCGAAUAUCCCUAAUGUGGAUAUCUCGGGCGUAUAAUUUUUGUUAGCCGGGACUGCGUUCGCGCUAUCCCGAAUAAAUAAUUCAAACAUAAUUAACGCUCGUUGAAAAAAACGAGUUUAUC

...........((((.((((..(((((..........))))).))))(((((.(.(((((..((..........))..)))))..))))))((((((((((....).)))))).))).)))).......xxxxxxxx(((((..(((....)))..))))).........................(((((((......))))))).....

>11_u1_pre2_[BX284605.5](https://www.ncbi.nlm.nih.gov/nuccore/BX284605.5)_14463352-14463189_Caenorhabditis_elegans_chromosome_V

AAACUUACCUGGCUGGGGGUUAUUUCGCGAUCAAGAAGGCGGAAUCCCCAUGGUGAGGCCUACCCAUUGCACUUUUGGGCGGGCUGACCUAUGUGGCAGUCUCGAGUUGAGAUUCGCCAACAGCUUAAUUUUUGCGUAUCGGGGCUGCGUGCGCGCGGCCCUGAAAAAAAGAUAUACAUUAAUGAUUUUGAAUCACUGCAACUAAUUUUUGGA

..........(((((((((..(((((((..........)))))))))))((((.(.(((((.((((.........)))).)))))..))))).((((((((((.....)))))).)))).)))))......xxxxxxx(((((((((((....)))))))))))..................(((((...)))))..................

>19_u1_pre2_[ABAV01021398.1](https://www.ncbi.nlm.nih.gov/nuccore/ABAV01021398.1)_79659-79494_Nematostella_vectensis_strain_CH2_x_CH6_NEMVEscaffold_262_Cont21398_whole_genome_shotgun_sequence.

AUACUUACCUGACGCGGGAGGUUUACCGUGAUCAUCAAGGCGGUCCUCUCAGGGCGAGGCCCUCUCAUUGCACUUCGAUUGGGUUGACCCUUGCGAUUACCCCAAAUGUGGGUAACUCGAGCGUAUAAUUUCUGGUAGUGGGGACCUGCGUUCGCGCUAGUCCCCGAACAAUUAACUAAUAGUCUCUCCUAAUUUAGCUAUAAUGCAACGGUUUU

......xxx..((((((((((...(((((..........)))))))))))((((...(((((..((..........))..)))))..))))..((((((((((....).)))))).))).))))........xxxx.((((((((..(((....)))..))))))..))..........(((......))).....((......)).........

**U2**

>19_u2_pre2_[CM000679_2](https://www.ncbi.nlm.nih.gov/nuccore/568336007)_43245864_43245674_Homo_sapiens_chromosome_17_GRCh38_reference_primary_assembly

AUCGCUUCUCGGCCUUUUGGCUAAGAUCAAGUGUAGUAUCUGUUCUUAUCAGUUUAAUAUCUGAUACGUCCUCUAUCCGAGGACAAUAUAUUAAAUGGAUUUUUGGAGCAGGGAGAUGGAAUAGGAGCUUGCUCCGUCCACUCCACGCAUCGACCUGGUAUUGCAGUACCUCCAGGAACGGUGCACCCCCUCCGGGGAUACAACGUGUUUCC

..(((((((..(((....)))..)))...))))......(((.....(.(...(((((((...((..((((((.....)))))).)))))))))x..)x)....xxxx)))((((.((((...((((....)))).))))))))..((((((.(((((.............)))))..)))))).((((....))))...x...........

>2_u2_pre2_[CM002897_2](https://www.ncbi.nlm.nih.gov/nuccore/1193823975)_18649682_18649492_Danio_rerio_chromosome_13_GRCz11_reference_primary_assembly

AUCGCUUCUCGGCCUUUUGGCUAAGAUCAAGUGUAGUAUCUGUUCUUAUCAGUUUAAUAUCUGAUACGUGCCCUACCCGGGCACCAUAUAUUAAAUUGAUUUUUGGAACAGGGAGAUGGAAUAGGGGCUUGCUCCGUCCACUCCACGCAUCGACCCGGUAUUGCAGUACCUCCGGGAACGGUGCACCCCCUAACCUGGUAAAAAAUAGAUUA

..(((((((..(((....)))..)))...))))......(((.....(..(((((((.((.((....((((((.....)))))))).))x)))))))xx)....xxxx)))((((.((((...((((....)))).))))))))..((((((.((((((((....)))))...)))..))))))....((......)).............. (-74)

>2_u2_pre2_[GG666467](https://www.ncbi.nlm.nih.gov/nuccore/GG666467)_1_968188_968378_Branchiostoma_floridae_genomic_scaffold_BRAFLscaffold_17_whole_genome_shotgun_sequence

AUCGCUUCUCGGCCUUUUGGCUAAGAUCAAGUGUAGUAUCUGUUCUUAUCAGUUUAAUAUCUGAUACGCUCCGCAUCGCGGAGCCAUAUAUUAAAUUGAUUUUUGGAAGGAGGCUAUGGACUAGGUGCUUGCACCAUCCUAGCCACGGGUUGGCCCGGUAUUGCAGUACCUCCGGGAUCGGCCCACCCCUCCGGGGGUUUAUCUAUAAUCUA

..(((((((..(((....)))..)))...)))).....((..........(((((((.((.((....(((((((...))))))))).))x)))))))xxx....xxx..))(((((.(((...((((....)))).))))))))..(((..((((((((((....)))))...))).))..))).((((....))))............... (-78.8)

>19_u2_pre2_[AABS01001062](https://www.ncbi.nlm.nih.gov/nuccore/AABS01001062)_1_345_536_Ciona_intestinalis_ciona1062_whole_genome_shotgun_sequence

AUCGCUUCAAGGCUAUUUUAGCUGUGAUCAAGUGUAGUACCUGUUCUUCUCAGGUUGAAAUCUGAGACGGAAACGAUUCGUUUCCUCUAUAUUUCAUUCGGAUUUUUGAACACACGGAAGGUAAUGAAGCUUGCUUCUACUUGCUCCGGGUUGUCCUGGUUUUGCAUUAUCGCCAGGUUCGGCCCACGUUCCACUUUGGUGGAUUUUAGUAUA

..(((((((..((((...))))..)))...))))..(((.............(..(.((((...(((.(((((((...))))))))))..))))x)..).xx.....xxxxx...((((((((..((((....)))))))))..)))(((..(.((((((...........))))))..)..)))....(((((....))))).....x))). (-59.6)

>4_u2_pre2_[AAGJ05082320](https://www.ncbi.nlm.nih.gov/nuccore/AAGJ05082320)_1_19260_19069_Strongylocentrotus_purpuratus_Contig82320_fixed_whole_genome_shotgun_sequence

AUCGCUUCUCGGCCUUUUGGCUAAGAUCAUGUGUAGUAUCUGUUCUUUUCAGCUUAAUAUCUGAAACGCGACUCACCGAGUCGCUUGUAUAUUAAACUGAUUUUUGAAUCUAGACCAUGGAAUAGGGGCUUGCUCCAUCCUGGUCACGGGUUGGCCCGGUAUUGCAGUACCUCCGGGAUCGGCCCACCCCUCAGGGGGUAAUAAUCGAACCAA

..(((.(((..(((....)))..)))....)))...........(...(.((.((((.((....((.(((((((...)))))))))..))x)))).))x).....)xxx...(((((.(((...((((....)))).))))))))..(((..(((((((.............))))).))..)))(((((....))))).............. (-70.2)

>12_u2_pre2_[AE014134](https://www.ncbi.nlm.nih.gov/nuccore/AE014134)_6_19815805_19815610_Drosophila_melanogaster_chromosome_2L

AUCGCUUCUCGGCCUUAUGGCUAAGAUCAAAGUGUAGUAUCUGUUCUUAUCAGCUUAACAUCUGAUAGUUCCUCCAUUGGAGGACAACAAAUGUUAAACUGAUUUUUGGAAUCAGACGGAGUGCUAGGGGCUUGCUCCACCUCUGUCACGGGUUGGCCCGGUAUUGCAGUACCGCCGGGAUUUCGGCCCAACUGAAUAAUAAAUAUUUAAUUAUAAA

..(((((((..(((....)))..)))....)))).................((.((((.((.((...((.(((((...)))))))..)).))x)))).))xxx....xxxxx..(((((((.....((((....))))..)))))))..(((..((((((((((....))...))))))...))..)))...((((((.....))))))........ (-63.5)

>5_u2_pre2_[EQ090210](https://www.ncbi.nlm.nih.gov/nuccore/EQ090210)_1_1404540_1404347_Anopheles_gambiae_M_scf_1925491382_genomic_scaffold_whole_genome_shotgun_sequence

AUCGCUUCUCGGCCUAAAGGCUAAGAUCAAAGUGUAGUAUCUGUUCUUAUCAGCUUAACAUCUGAUAGCUCUCCCAUAGGGAGACAACAAAUGUUAAACUGAUUUUUGGCAAGGGGAGGAAAGUUCGGGGCUUGCUCCACUUCUUCCGCGGGUUGGCCCGGUAUUGCAGUACCGCCGGGAUCGGCCCACAUUCAUUCUAUUAAACAAAAAUUGUG

..(((((((..(((....)))..)))....)))).................((.((((.((.((.....((((((...))))))...)).))x)))).))xxx.....xxx...(((((((.((..((((....)))))))))))))..(((..((((((((...........)))))).))..)))................((((...)))). (-65.2)

>14_u2_pre2_[CM000061](https://www.ncbi.nlm.nih.gov/nuccore/CM000061)_5_7231897_7232087_Apis_mellifera_strain_DH4_linkage_group_8_whole_genome_shotgun_sequence

AUCGCUUCUCGGCCUGAUGGCUAAGAUCAAAGUGUAGUAUCUGUUCUUAUCAGCUUAAUAUCUGGUACACUCCCCACCGGGAGUCAGAAUAUUAGUCUGAUUUUUGGAACCGGGCGGAACCCCGGGGCUUGCUCCGCUUCUGCCGCGAGUCGGCUCGGCAUUGCAGUGCCGUCGAGAUCGGCUCAAUAAGUUACCCCAAAGAUUAAAGAAUU

..(((((((..(((....)))..)))....)))).....(.(..((.....((..(((.(((((....((((((....))))))))).))x)))..))xxx.xxxxx.....(((((((...(((((....))))).)))))))..(((((((((((((((....)))))...))).)))))))................))....)x)... (-72)

>2_u2_pre2_[GL341007](https://www.ncbi.nlm.nih.gov/nuccore/GL341007)_1_168927_169119_Nasonia_vitripennis_chromosome_4_unlocalized_genomic_scaffold_Chr4_random066_whole_genome_shotgun_sequence

AUCGCUUCUCGGCCUAUUGGCUAAGAUCAAAGUGUAGUAUCUGUUCUUAUCAGCUUGAUAUCUGAUACGCUCCUCAUUGAGGAGCCAGAAUAUCGAACUGAUUUUUGGAAUAUGGCGGAGUGUCUGGGGCUUGCUCCGACUCCGCCACGGGUCGACCUUGCAUUGCAGUACCGCAAGGAACGGCCCACACAAUUAAUUCAAACAAAAUCUUCUC

..(((((((..(((....)))..)))....)))).......((((......((.((((.(((((....(((((((...)))))))))).))x)))).))xxx....xx)))).((((((((...(((((....)))))))))))))..((((((.((((((...........))))))..))))))............................ (-79.3)

>1_u2_pre2_[BX284601](https://www.ncbi.nlm.nih.gov/nuccore/BX284601)_5_12324944_12324754_Caenorhabditis_elegans_chromosome_I

AUCGCUUCUUCGGCUUAUUAGCUAAGAUCAAAGUGUAGUAUCUGUUCUUAUCGUAUUAACCUACGGUAUACACUCGAAUGAGUGUAAUAAAGGUUAUAUGAUUUUUGGAACCUAGGGAAGACUCGGGGCUUGCUCCGACUUCCCAAGGGUCGUCCUGGCGUUGCACUGCUGCCGGGCUCGGCCCAGUCCCCGAGGGGACAAAAUAAGCUUAC

..((((((((.((((....))))))))....))))........(((......(((..(((((...((.(((((((....)))))))))..)))))x)))xxx....xx)))...((((((..((((((....))))))))))))..((((((.((((((((......)).))))))..)))))).(((((....)))))............. (-82.1)

>5_u2_pre2_[ABAV01032332](https://www.ncbi.nlm.nih.gov/nuccore/ABAV01032332)_1_475_283_Nematostella_vectensis_strain_CH2_x_CH6_NEMVEscaffold_729_Cont32332_whole_genome_shotgun_sequence

AUCGCUUCUCGGCCUUUUGGCUAAGAUCAAGUGUAGUAUCUGUUCUUAUCAGCUUAAUAUCUGAUACGCUGCUCAUUGAGUAGCUCAUAUAUUAAACUGAUUUUUGGAAACUGGCUGUGGAAUAAGCGGCUUGCUGCGUCCCAGCCACGGGUUGUCUCGGUAUUGCACUACCUCCGAGUACGGCCCCCUUCCCUUUCGGGAAGACACAUUCAAG

..(((((((..(((....)))..)))...)))).........((......((.((((.((.(((...(((((((...)))))))))).))x)))).))xxx....xx))..((((((.(((...(((.....)))...))))))))).(((..(((((((.............))))).))..))).((((((....))))))........... (-75.5)

**U4**

>2_[u4_pre_hs](https://www.ncbi.nlm.nih.gov/nucleotide/M15956.1?report=genbank&log$=nuclalign&blast_rank=1&RID=G5VF69P401R&from=271&to=421)

AGCUUUGCGCAGUGGCAGUAUCGUAGCCAAUGAGGUCUAUCCGAGGCGCGAUUAUUGCUAAUUGAAAACUUUUCCCAAUACCCCGCCGUGACGACUUGCAAUAUAGUCGGCACUGGCAauuuuugACAGUCUCUACGGAGACUGAAUUUUU

.........(((((((((((((((.(((.....((.....))..))))))).))))))).))))...........x((......((((((.(((((........))))).))).))).....))..(((((((....)))))))....... (-47)

>2_[u4_pre_Dr](https://www.ncbi.nlm.nih.gov/nucleotide/LR812087.1?report=genbank&log$=nuclalign&blast_rank=1&RID=G5VGUNAM01R&from=5956351&to=5956499)

AGCUUUGCGCAGUGGCAGUAUCGUAGCCUAUGAGGUUUAUCCGAGGCGCGAUUAUUGCUAAUUGAAAACUUUACCCAAUACCCCGCCGUGACGACUUGAAAUAUAGUCGGCACUGGCAauuuuugACAGUCUUCUCGAAGACUGAAAUA

.........(((((((((((((((.((((..((......))..)))))))).))))))).))))...........x((......((((((.(((((........))))).))).))).....))..(((((((....)))))))..... (-44)

>10_u4_pre_[GG666612](https://www.ncbi.nlm.nih.gov/nuccore/GG666612)_1_8898552_8898412_Branchiostoma_floridae_genomic_scaffold_BRAFLscaffold_196__whole_genome_shotgun_sequence_

AGCUUUGCGCAGAGGCGAUAUCAUAGCCAAUGAGGUCCAACCGAGGCGUGAUUAUUGCUAGUUGAAAACUUUUCCCAAUACCCCGCCUGGGGGACGUGAAAUACCGUCCACUAUGGCAauuuuugUGAGCCCCUACGGGGGCCCCCAU

.((.....))...(((((((((((.(((.....((.....))..))))))).))))))).............x(.(((......((((((.(((((........))))).))).))).....))).).(((((....)))))...... (-44.2)

>3_u4_[AABS01000396](https://www.ncbi.nlm.nih.gov/nuccore/AABS01000396)_1_28948_28808_Ciona_intestinalis_ciona0396__whole_genome_shotgun_sequence_

AGCUUUGCGCAGUGGCGGUAUCGUAGCUGAUGAGGUUUAUCCGAGGCGCGAUUAUUGCUAGUUGAAAACUAUUACCAAUACCCCGCCCUGUCGACGUGAAAAACCGUCGACUGUGGCAauuucugAUGGGUUUUCCGGAACAUUUCCU

.((.....))..((((..((((((.(((.....((.....))..))))))).))..))))...x((((xx((((..(((...((((...(((((((........))))))).))))..)))..))))...))))..((((...)))). (-38.1)

>1_u4_pre_[AAGJ05065406](https://www.ncbi.nlm.nih.gov/nuccore/AAGJ05065406)_1_6450_6590_Strongylocentrotus_purpuratus_Contig65406_fixed__whole_genome_shotgun_sequence_

AUCUUUGCGGAGAGGCAGUAUCGUAGCUAAUGAGGUUUAUCCGAGGCGCGAUUAUUGCUAGUUGAAAACUUAUCCCAAUACCCCGCUCUGACGACGUGAAAUACCGUGGGCUGUAGCAauuugUCCAGAUCUCCAAGGAGAUCUUACA

......((((...(((((((((((.(((.....((.....))..))))))).))))))).((((...........))))...))))...xxx((..((..(((((...)).)))..))..))....(((((((....))))))).... (-36.8)

>2_u4_pre_[AE014134](https://www.ncbi.nlm.nih.gov/nuccore/AE014134)_6_21215178_21215039_Drosophila_melanogaster_chromosome_2L

AUCUUUGCGCAGUGGCAAUACCGUAACCAAUGAAGUCCUCCUGAGGUGCGGUUAUUGCUAGUUGAAAACUUUAACCAAUACCCCGCCAUGGGGACGUGAAAUACCGUCCACUACGGCAauuuuugGAAGCCCGAGAGGGCUAAUUAAAUA

.........(((((((((((((((.(((.....((.....))..)))))))).))))))).)))..........x(((......(((.((((((((........))))).))).))).....)))..(((((....)))))......... (-46.1)

>1_u4_pre_[KB671676](https://www.ncbi.nlm.nih.gov/nuccore/KB671676)_1_581842_581982_Anopheles_epiroticus_strain_epiroticus2_unplaced_genomic_scaffold_supercont1_45__whole_genome_shotgun_sequence_

AGCUUUGCGCAGUGGCGAUAUCGUAACCAAUGAGGUACAACCGAGGUGCGAUUAUUGCUAGUUGAAAACUAAUACCAAUACCCCGCCUUGGGGACGUGAAAUACCGUCCGCUAUGGCAauuuuugGAAACCCCGAAAGGGGUCAUAAU

.........((((((((((((((((.((.....((.....))..))))))).)))))))).)))..........x(((......(((.((((((((........))))).))).))).....)))...(((((....)))))...... (-47.6)

>2_u4_pre_[CM000054](https://www.ncbi.nlm.nih.gov/nuccore/CM000054)_5_13590219_13590079_Apis_mellifera_strain_DH4_linkage_group_1__whole_genome_shotgun_sequence_

AGCUUUGCGCAGUGGCGAUAUCGUAACCAAUGAGGUUCUACCGAGGUGCGAUUUUUGCUAGUUGAAAACUUUUACCAAUACCCCGCCAUGACGAUGUGAAAAAUCAUCGGCUACGGCAauuuuugGUAACCCCUACGGGGGUUUUUAA

.........(((((((((.((((((.((.....((.....))..))))))))..)))))).)))........xxx(((......(((.((.(((((........)))))..)).))).....)))...(((((....)))))...... (-42.1)

>2_[u4_pre_Nvi](https://www.ncbi.nlm.nih.gov/nucleotide/XR_004227174.1?report=genbank&log$=nuclalign&blast_rank=1&RID=G5XS94D901R&from=1&to=141)

AUCUCUGCGCAGUGGCGAUAUCGUAACCAAUGAGGUUCUACCGAGGUGCGAUUAUUGCUAGUUGAAAACUUUUACCAAUACCCCGCCAAGACGAUGUGAAAUACCAUCGGCUACGGCAauuuuugACAGCCCUUACGAGGGUUAUAUU

.........((((((((((((((((.((.....((.....))..))))))).)))))))).)))...........x((......(((.((.(((((........))))).))..))).....))...((((((....))))))..... (-34.7)

>5_[u4_pre_Ce](https://www.ncbi.nlm.nih.gov/nucleotide/CP038191.1?report=genbank&log$=nuclalign&blast_rank=1&RID=G5XU91XX016&from=11518735&to=11518888)

AGCUUUGCGCUGGGGCGAUAACGUGACCAAUGAGGCUUUGCCGAGGUGCGUUUAUUGCUGGUUGAAAACUUUUCCCAAUUGCCCGCGAUGUCCCCUGAAACAUGGGUGGCAUACGCAauuuuugAAAGCCUCUAGGAGGCAGAAAAACAUCUUC

(((.....)))..(((((((((((.(((.....(((...)))..)))))).))))))))..........x((((..........(((((((((((........))).))))).)))...........(((((...))))).))))......... (-42.5)

>7_u4_pre_[ABAV01042195](https://www.ncbi.nlm.nih.gov/nuccore/ABAV01042195)_1_334_472_Nematostella_vectensis_strain_CH2_x_CH6_NEMVEscaffold_2534_Cont42195__whole_genome_shotgun_sequence_

AGCUUUGCGCAGUGGCAUUACCGUAGCUGAUGAGGUCCAUCCGACGCGCGGUUAUUGCUGAUUGAAAACUUUUCCCAAUACCCCGCGAGACGACUUGAAACAUAGUCGGCUUUGCAauuuuugUGGGCCCUAUUUAGGGCCACCAU

.........((((((((..(((((.((.((((.....))))....)))))))...)))).))))...........x((......(((((.(((((........)))))..))))).....))...((((((....))))))..... (-40)

**U5**

>16_u5_pre5_[CM000677](https://www.ncbi.nlm.nih.gov/nuccore/CM000677)_2_65296051_65296166_Homo_sapiens_chromosome_15_GRCh38_reference_primary_assembly

AUACUCUGGUUUCUCUUCAGAUCGCAUAAAUCUUUCGCCUUUUACUAAAGAUUUCCGUGGAGAGGAACAACUCUGAGUCUUAACCCAAUUUUUUGAGGCCUUGCUUUGGCAAGGCUAUAUGUGGUAAUCCAACAAUAGAAAUUAUUUUUAAGUUUGUGUGUUCC

..((((..(((((((((((...((...((((((((...........)))))))).)))))))))))))......))))....xxxxxxx.......((((((((....))))))))..............((((.((.(((((.......))))).)))))).. (-35.9)

>10_u5_pre5_[CM002889](https://www.ncbi.nlm.nih.gov/nuccore/CM002889)_2_43123291_43123176_Danio_rerio_chromosome_5_GRCz11_reference_primary_assembly

CAGCUCGAGUUUCUCUUCAAACACGCACAAAUCUUUCGCCUUUUACUAAAGAUUUCCGUGGGGAGGAACUAUUGUGAGUUAUGUUUAUUUUUGGGUGCUCUGCUAUCUGCAGAGCUGCAUUAAGAUGUUGAAUUGCAGAACGAGAUGUGGAAGAGUGUUUGUGU

.((((((((((((((..(....(((...((((((((...........)))))))).))))..))))))))....)))))).....xxxxxx...(((((((((.....))))))).))................(((((((.............)).))))).. (-38.8)

>8_u5_pre5_[GG666485](https://www.ncbi.nlm.nih.gov/nuccore/GG666485)_1_1370472_1370594_Branchiostoma_floridae_genomic_scaffold_BRAFLscaffold_37_whole_genome_shotgun_sequence

UCACUCUGGUUUCCCUUCAAUCCACCACGCACAAAUCUUUCGCCUUUUACUAAAGAUUUCCGUGGAGGGGAACAAUCAAUGAGUCUAUAAACAAUUUUUCUCUGCCCUGCCUUGUGCAGGGCUCCUAAUAAACCAAAGAAGUCCACACCCAACACACCUUUGCUAAGCCUA

..((((..((((((((........(((((...((((((((...........)))))))).))))))))))))).......)))).........xxx.......(((((((.....)))))))...........(((((..((...........))..)))))......... (-35.5)

>9_u5_pre5_[AABS01000196](https://www.ncbi.nlm.nih.gov/nuccore/AABS01000196)_1_104341_104227_Ciona_intestinalis_ciona0196_whole_genome_shotgun_sequence

CCACUCUGGCUUCUCUUCAGCGCAUGAUUCUUUCGCCUUUUACUAAAGAAUUCCGUGGAGGGGAGCAUUUCAAUGAGUCUUUAACAAUUUUUCUUUACCCGAUUUCGGUCGGGCUCCUAUUUUUAACCAUUUUUUUAUAUGUAUCGAUUACGAGCUGUAAAUG

..((((..(((((((((((.((...((((((((...........)))))))).)))))))))))))........))))..xxxx.(((.........((((((....)))))).....)))............((((((.((.(((....))))))))))).. (-32.6)

>17_u5_pre5_[AAGJ05103420](https://www.ncbi.nlm.nih.gov/nuccore/AAGJ05103420)_1_12808_12925_Strongylocentrotus_purpuratus_Contig103420_fixed_whole_genome_shotgun_sequence

AUGGGUGGUUUUUCUGCAAUACGCAUAAAUCUUUCGCCUUUUACUAAAGAUUUCCGUGCAGAGGAACACAUUGAUGAGUCUAUAAAGAUUUUUCUCUGGCUUAAAAUUAUUUGAUGCAAAGGAAUUCAAUCGAUCGAUGAACAGGCUCUGUCCUACCAACAUCGAU

((..((((((..(((((...(((...((((((((...........)))))))).))))))))..))).)))..))...........xxxxxxx((...(((((((....))))).))..))...........(((((((...(((......))).....))))))) (-32.5)

>2_u5_pre5_[AE014134](https://www.ncbi.nlm.nih.gov/nuccore/AE014134)_6_19812075_19811954_Drosophila_melanogaster_chromosome_2L

UUACUCUGGUUUCUCUUCAAUUGUCGAAUAAAUCUUUCGCCUUUUACUAAAGAUUUCCGUGGAGAGGAACACUCUAAUGAGUCUAAACUCAAUUUUUGUAUGACCUGGCUAAAUAUUUAGUUGGGCCAAUGAAUAAUAAAUAAAAAUGAACGAAAUCCGUUCGAUGAAGG

........(((((((((((.....((...((((((((...........)))))))).))))))))))))).......(((((....)))))...xx..(.((.((..(((((....)))))..)).)))..................(((((.....)))))........ (-36.2)

>5_u5_pre5_[CM000359](https://www.ncbi.nlm.nih.gov/nuccore/CM000359)_1_15809445_15809564_Anopheles_gambiae_str_PEST_chromosome_3R_whole_genome_shotgun_sequence

GCACUCUGAUCUCUCUUCAACUGUCGAAUAAAUCUUUCGCCUUUUACUAAAGAUUUCCGUGGAGAGGGAUACUCUAAUGAGUCUAUAGUGAAUUUUUGUCCGUCUCGAUUCCGUAAGGAGUCGAGCCCUAAACUUCAAUACAAAAAACUUAUAGCAUAAAUCUGUGGA

.(((....(((((((((((.....((...((((((((...........)))))))).)))))))))))))......xxxxxx......xxxx.(((.....(.(((((((((....))))))))))...)))...............................))).. (-39.4)

>20_u5_pre5_[CM000059](https://www.ncbi.nlm.nih.gov/nuccore/CM000059)_5_9966625_9966505_Apis_mellifera_strain_DH4_linkage_group_3_whole_genome_shotgun_sequence

GUACUCUGGUUUCCCUUCAAAUCACGCAUAAAUCUUUCGCCUUUUACUAAAGAUUUCCGUGGAGGGGAACAAUUGAUGAGUCUAUAGACUAAUUUUUUGUAUACCCGGCGCAAGCUGGGUCAUUAAUAAAAUAAAACAUAAUUAGUAAUUUAUUUUUAUAUUUGUGUAU

..((((..(((((((((.....((((...((((((((...........)))))))).))))))))))))).......))))..........xxxxxxxx...(((((((....)))))))...............((((((...((((.......))))..)))))).. (-40.7)

>5_u5_pre5_[CM000918](https://www.ncbi.nlm.nih.gov/nuccore/CM000918)_2_9172767_9172648_Nasonia_vitripennis_chromosome_4_whole_genome_shotgun_sequence

UUACUCUGGUUUCCCUUCAUUACACGCAUAAAUCUUUCGCCUUUUACUAAAGAUUUCCGUGGAGGGGAACACUUGAUGAGUCUAUAAAAAAUUUUUUGUGUGCCCGACGAAAGUUGGGCCGUUAUAUAAUUUAAAAAUAAUUCGACCUAGUGAUCGAUCACGUAAUCG

..((((.(((((((((((....((((...((((((((...........)))))))).)))))))))))).)))....)))).........xxxxxxxx((((((((((....)))))))....)))................(((....((((....))))....))) (-41.6)

>8_u5_pre5_[BX284604](https://www.ncbi.nlm.nih.gov/nuccore/BX284604)_4_9444641_9444762_Caenorhabditis_elegans_chromosome_IV

UAACUCUGGUUCCUCUGCAUUUAACCGUGAAAAUCUUUCGCCUUUUACUAAAGAUUUCCGUGCAAAGGAGCAUACAUUGAGUAUUAUAUACAAUUUUUGGAGUCCCCUUGAGAAAGCGGGACAAAAGUUUGGAGCAGUAUUACACGAUAUGAAUGCAAAUUCAUUUAUUG

..((((..((((((.((((......((.(.((((((((...........))))))))))))))).)))))).......)))).....((((...xxx..((((((((((....))).))))).....)).......))))....((((((((((....)))))..))))) (-35.9)

>6_u5_pre5_[ABAV01015157](https://www.ncbi.nlm.nih.gov/nuccore/ABAV01015157)_1_376_255_Nematostella_vectensis_strain_CH2_x_CH6_NEMVEscaffold_140_Cont15157_whole_genome_shotgun_sequence

GCACUCUGGUUUUCCUCCAUAUCGAGUAAAUCUUUCGCCUUUUACAAAAGAUUUCCGUCGAGGAGAGCACUGAAAUGAGUAUAUCACUCAAUUUUUGAUUUGCCCUGCAUUUUUGCGGGGCUUACACACUAAAUGAAAAAUUGCGGCCCAAAUGGUUCAACUGAAUUUUG

..((((..(((((((((.....((...((((((((...........)))))))).))..)))))))))........))))......((((xxxxxx.((((((((((((....)))))))).........)))).......)).))........((((....)))).... (-39.9)
