# Supplementary material for: The SMN complex drives structural changes in human snRNAs to enable snRNP assembly
Source: Nat Commun. 2023 Oct 18;14:6580. doi: 10.1038/s41467-023-42324-0 (PMC10584915; doi:10.1038/s41467-023-42324-0)
Supplement: Supplementary file 5 — Supplementary Data 2 [file 41467_2023_42324_MOESM5_ESM.zip › Supplementary Data-2.docx]

**Supplementary Data 2**

Constraints used to model structure changes of folding interproducts leading to final structures for individual species of individual pre-snRNAs via the constrained secondary structure prediction. The files contain sequences of individual pre-snRNAs in fasta format, complemented with the constraint in dot-bracket format. The sequences together with their constraints were the input to the secondary structure prediction algorithm that was RNAsubopt –C. For hyperlinks of genomic IDs , please see Supplementary Data 1.

**U1**

>6_14_u1_pre2_GG666660.1_4494-4657_Branchiostoma_floridae_genomic_scaffold_BRAFLscaffold_244_whole_genome_shotgun_sequence.

AUACUUACCUGGCAGAGAUGGCACAUUGAUCACGAAGGAUGUCCGUCCAGGGCGAGGCCCGACCAUUGCACUCCGGUUGGGUUGACCCUUGCGAUUACCCCAAAUGCGGGUAACUCGGCUGCGUAAUUUAUGAUAGUGGGGGUCUGCGUUCGCGCUAACCCCCGCCUUUACAAUCCAAAAGCAAGUAUACAACACACCUGAUCAUAUCCGACA

xxxx.......((((.(((((.(((((..........))))))))))(((((...(((((((((..........)))))))))...)))))((((((((((....).)))))).))).))))...xxxxxxx...((((((((..(((....)))..))))))))................................................

>45_9_u1_pre2_CM002888.2_69962711-69962548_Danio_rerio_chromosome_4_GRCz11_reference_primary_assembly.

AUACUUACCUGGCAGGGGAGAUACCAUGAUCAAGAAGGUGGUUCACCCAGGGCGAGGCUUGGCCAUUGCACUCCGGCCACGCUGACCCCUGCGAAUUCCCCAAAUGUGGGAAUCUCGACUGCAUAAUUUCUGGUAGUGGGGGACUGCGUUUGCGCUCUCCCCUGAGCACAGUGGUUCAAGACAGAGUUCAGGCGGUAGCGUCGGACCCGGCUG

xxxxxxxxxxxxxx.....((.(((((..........)))))))...(((((...(((.(((((..........))))).)))...)))))((((((((((....).)))))).))).......xxxxxxxxx....((((((..(((....)))..))))))((((......)))).............((((....))))...xxx.....

>25_13_u1_28_1_pre2_XR_007067098.1_PREDICTED_Homo_sapiens_U1_spliceosomal_RNA_LOC124904616_ncRNA

AUACUUACCUGGCAGGGGAGAUACCAUGAUCACGAAUGUGGUUUUCCCAGGGCGAGGCUUAUCCAUUGCACUCCGGAUGUGCUGACCCCUGCGAUUUCCCCAAAUGUGGGAAACUCGACUGCAUAAUUUGUGGUAGUGGGGGACUGCGUUCGCGCUUUCCCCUGGGUGAGAUCACCCCACAUAAUUUAUUCUAAAAUGUAUUUACUUACAUGU

...............((((((.(((((..........)))))))))))((((...(((.(((((..........))))).)))...)))).((((((((((....).)))))).)))........xxxxxxx.....((((((..(((....)))..))))))(((((....)))))..................(((((......)))))..

>18_6_u1_pre2_AABS01000073.1_3124-2961_Ciona_intestinalis_ciona0073_whole_genome_shotgun_sequence.

AUACUUACCUGACGAGGGCCUUCACUGCGAUCAGGCAGGCAGUGGGUCCAAGGUGAGGCCAGGCCAUUGCACAUCGGCUUAGCUGACCCUUGCGAUUACCCUAAAUGUUGGUAACUCGGUCGUAUAAUUUCUGCUAGUGGGGACUGCGUUCGCGCUAUCCCCGCCCCAAUCUUUAAUCCUAGUUUAGCAAAACAAGUACAUGAACUUGUGUAC

...........(((((((((..((((((..........)))))))))))((((...(((.(((((..........))))).)))...)))).(((((((((.....)..))))).))).))))...xxxxxxx.....(((((..(((....)))..))))).............................((((((......))))))....

>4_7_u1_pre2_AAGJ05083895.1_7779-7940_Strongylocentrotus_purpuratus_Contig83895_fixed_whole_genome_shotgun_sequence.

AUACUUACCUGGCGCAGGGGUCGCAUUGAUCAAGAAGGAUGCACCCCCAGGGCGAGGCUUGCCAUUGCACUCCGGCUUGCUGAAUCUUGCGAUUCCCCCAAACGUGGGGAACUCGGGCGUAUAAUUUAUGGUAGCGGAGAUCUGCGUUCGCGCUAUCUCCUACAAUUCAAAAUGCAAAGAAAGAAAAACAUCGAAAAAUAUCAUGACAUUU

...........((((.(((((.(((((..........))))))))))(((((...(((..(((..........)))..)))...)))))((((((((((....).)))))).))).))))...xxxxxxx.....((((((..(((....))).))))))...................................................

>23_13_u1_pre2_AE014297.3_23826334-23826498_Drosophila_melanogaster_chromosome_3R.

AUACUUACCUGGCGUAGAGGUUAACCGUGAUCACGAAGGCGGUUCCUCCGGAGUGAGGCUUGGCCAUUGCACCUCGGCUGAGUUGACCUCUGCGAUUAUUCCUAAUGUGAAUAACUCGUGCGCGUAAUUUUUGGUAGCCGGGAAUGGCGUUCGCGCCGUCCCGACAUUUAAAAUAAAUAUUGAAAAGAAAACGACUCAACUUUAGUUAAUAAUU

...........(((((((((..((((((..........))))))))))(((((...(((((((((..........)))))))))...)))))((((((((((....).)))))).))))))))...xxxxxxx...(.(((((.(((((....)))))))))).).................................................

>23_14_u1_pre2_EQ090204.1_7076076-7075911_Anopheles_gambiae_M_scf_1925491376_genomic_scaffold_whole_genome_shotgun_sequence.

AUACUUACCUGGCACAGGGGUUACCGUGAUCACGAAGGCGGUUCCCCCAGGGCGAGGCCUGGCCAUUGCACACUUAGGCUGGGUUGACCCCUGCGAUUAUCCCUCAUGUGGAUAACUCGUGUGCGCAAUUUUUGGUAGCCGGGAAGUGCGUCCGCGCACAUCCCGAUUUAAUCUGAAAUACAAAACAAAUAUAUGUCAAGUUUUAACUUUUUACA

...........(((((((((..(((((..........))))).))))(((((...(((((((((............)))))))))...)))))((((((((((....).)))))).))))))))...xxxxxxx.....(((((.(((((....))))).)))))..................................................

>7_6_u1_pre2_CM000069.5_7012443-7012279_Apis_mellifera_strain_DH4_linkage_group_16_whole_genome_shotgun_sequence.

AUACUUACCUGACGCAGAGGUUACCGUGAUCAUAAAGGCGGUUCCUCCAGGGCGAGACUCGUCCAUUGCACUUGUGGAUCUGAGCUGACCCCUGCGAAUGCCCCUAAUGCGGGUGUCUCGGGCGUAAAAUUUUUGGUAGUCGGGACUGCGUUCGCGCUAUCCCGCGAAUAUUAAUUAAAAAAAUAAUAAUAAUAUAACAUUCUCCAAUUGGAAA

...........((((.((((..(((((..........))))).))))(((((..((.(((((((((.......))))))..)))))...)))))((((((((((....).)))))).))).))))...xxxxxxx.....(((((..(((....)))..)))))......................................(((....)))..

>3_19_u1_pre2_CM000916.2_26139276-26139115_Nasonia_vitripennis_chromosome_2_whole_genome_shotgun_sequence.

AUACUUACCUGGCGCAGAGGCUACCGUGAUCACGAAGGCGGUUCCUCCAGGGCGAGGCUCUUCCAUUGCACUACGGUCGAGCUGACCCUUGCGAAUAUCCCUAAUGUGGAUAUCUCGGGCGUAUAAUUUUUGUUAGCCGGGACUGCGUUCGCGCUAUCCCGAAUAAAUAAUUCAAACAUAAUUAACGCUCGUUGAAAAAAACGAGUUUAUC

...........((((.((((..(((((..........))))).))))(((((...(((((..((..........))..)))))...)))))((((((((((....).)))))).))).))))...xxxxxxx.....(((((..(((....)))..))))).........................(((((((......))))))).....

>37_11_u1_pre2_BX284605.5_14463352-14463189_Caenorhabditis_elegans_chromosome_V

AAACUUACCUGGCUGGGGGUUAUUUCGCGAUCAAGAAGGCGGAAUCCCCAUGGUGAGGCCUACCCAUUGCACUUUUGGGCGGGCUGACCUAUGUGGCAGUCUCGAGUUGAGAUUCGCCAACAGCUUAAUUUUUGCGUAUCGGGGCUGCGUGCGCGCGGCCCUGAAAAAAAGAUAUACAUUAAUGAUUUUGAAUCACUGCAACUAAUUUUUGGA

..........(((((((((..(((((((..........)))))))))))((((...(((((.((((.........)))).)))))...)))).((((((((((.....)))))).)))).)))))..xxxxxxx....(((((((((((....)))))))))))..................(((((...)))))..................

>3_19_u1_pre2_ABAV01021398.1_79659-79494_Nematostella_vectensis_strain_CH2_x_CH6_NEMVEscaffold_262_Cont21398_whole_genome_shotgun_sequence.

AUACUUACCUGACGCGGGAGGUUUACCGUGAUCAUCAAGGCGGUCCUCUCAGGGCGAGGCCCUCUCAUUGCACUUCGAUUGGGUUGACCCUUGCGAUUACCCCAAAUGUGGGUAACUCGAGCGUAUAAUUUCUGGUAGUGGGGACCUGCGUUCGCGCUAGUCCCCGAACAAUUAACUAAUAGUCUCUCCUAAUUUAGCUAUAAUGCAACGGUUUU

......xxx..((((((((((...(((((..........)))))))))))((((...(((((..((..........))..)))))..))))..((((((((((....).)))))).))).))))...xxxxxxx.....((((((..(((....)))..))))))...............................((......)).........

**U2**

>7_19_u2_pre2_CM000679_2_43245864_43245674_Homo_sapiens_chromosome_17_GRCh38_reference_primary_assembly

AUCGCUUCUCGGCCUUUUGGCUAAGAUCAAGUGUAGUAUCUGUUCUUAUCAGUUUAAUAUCUGAUACGUCCUCUAUCCGAGGACAAUAUAUUAAAUGGAUUUUUGGAGCAGGGAGAUGGAAUAGGAGCUUGCUCCGUCCACUCCACGCAUCGACCUGGUAUUGCAGUACCUCCAGGAACGGUGCACCCCCUCCGGGGAUACAACGUGUUUCC

..(((((((.((((....)))).)))...))))..................................((((((.....))))))..............xxxxxxxxxxx..((((.((((...((((....)))).))))))))..((((((.(((((.............)))))..)))))).((((....))))...x...........

>6_2_u2_pre2_CM002897_2_18649682_18649492_Danio_rerio_chromosome_13_GRCz11_reference_primary_assembly

AUCGCUUCUCGGCCUUUUGGCUAAGAUCAAGUGUAGUAUCUGUUCUUAUCAGUUUAAUAUCUGAUACGUGCCCUACCCGGGCACCAUAUAUUAAAUUGAUUUUUGGAACAGGGAGAUGGAAUAGGGGCUUGCUCCGUCCACUCCACGCAUCGACCCGGUAUUGCAGUACCUCCGGGAACGGUGCACCCCCUAACCUGGUAAAAAAUAGAUUA

..(((((((.((((....)))).)))...))))..................................((((((.....))))))..............xxxxxxxxxxx..((((.((((...((((....)))).))))))))..((((((.((((((((....)))))...)))..))))))....((......)).........x....

>8_2_u2_pre2_GG666467_1_968188_968378_Branchiostoma_floridae_genomic_scaffold_BRAFLscaffold_17_whole_genome_shotgun_sequence

AUCGCUUCUCGGCCUUUUGGCUAAGAUCAAGUGUAGUAUCUGUUCUUAUCAGUUUAAUAUCUGAUACGCUCCGCAUCGCGGAGCCAUAUAUUAAAUUGAUUUUUGGAAGGAGGCUAUGGACUAGGUGCUUGCACCAUCCUAGCCACGGGUUGGCCCGGUAUUGCAGUACCUCCGGGAUCGGCCCACCCCUCCGGGGGUUUAUCUAUAAUCUA

..(((((((.((((....)))).)))...))))..................................(((((((...)))))))..............xxxxxxxxxxxx.(((((.(((...((((....)))).))))))))..(((((((((((((((....)))))...))).)))))))(((((....)))))...........x..

>4_19_u2_pre2_AABS01001062_1_345_536_Ciona_intestinalis_ciona1062_whole_genome_shotgun_sequence

AUCGCUUCAAGGCUAUUUUAGCUGUGAUCAAGUGUAGUACCUGUUCUUCUCAGGUUGAAAUCUGAGACGGAAACGAUUCGUUUCCUCUAUAUUUCAUUCGGAUUUUUGAACACACGGAAGGUAAUGAAGCUUGCUUCUACUUGCUCCGGGUUGUCCUGGUUUUGCAUUAUCGCCAGGUUCGGCCCACGUUCCACUUUGGUGGAUUUUAGUAUA

..(((((((.((((.....)))).)))...))))..............................(((.(((((((...)))))))))).............xxxxxxxxxx....((((((((..((((....)))))))))..)))((((((.((((((...........))))))..))))))....(((((....))))).....x....

>10_4_u2_pre2_AAGJ05082320_1_19260_19069_Strongylocentrotus_purpuratus_Contig82320_fixed_whole_genome_shotgun_sequence

AUCGCUUCUCGGCCUUUUGGCUAAGAUCAUGUGUAGUAUCUGUUCUUUUCAGCUUAAUAUCUGAAACGCGACUCACCGAGUCGCUUGUAUAUUAAACUGAUUUUUGAAUCUAGACCAUGGAAUAGGGGCUUGCUCCAUCCUGGUCACGGGUUGGCCCGGUAUUGCAGUACCUCCGGGAUCGGCCCACCCCUCAGGGGGUAAUAAUCGAACCAA

..(((.(((.((((....)))).)))....)))..................................(((((((...)))))))...............xxxxxxxxxxx..(((((.(((...((((....)))).))))))))..((((((((((((.............))))).)))))))(((((....))))).......x......

>6_12_u2_pre2_AE014134_6_19815805_19815610_Drosophila_melanogaster_chromosome_2L

AUCGCUUCUCGGCCUUAUGGCUAAGAUCAAAGUGUAGUAUCUGUUCUUAUCAGCUUAACAUCUGAUAGUUCCUCCAUUGGAGGACAACAAAUGUUAAACUGAUUUUUGGAAUCAGACGGAGUGCUAGGGGCUUGCUCCACCUCUGUCACGGGUUGGCCCGGUAUUGCAGUACCGCCGGGAUUUCGGCCCAACUGAAUAAUAAAUAUUUAAUUAUAAA

..(((((((.((((....)))).)))....)))).................................((.(((((...)))))))................xxxxxxxxxxxx.(((((((.(...((((....)))).))))))))..(((((((((((((...........))))))...)))))))...((((((.....))))))....x...

>4_5_u2_pre2_EQ090210_1_1404540_1404347_Anopheles_gambiae_M_scf_1925491382_genomic_scaffold_whole_genome_shotgun_sequence

AUCGCUUCUCGGCCUAAAGGCUAAGAUCAAAGUGUAGUAUCUGUUCUUAUCAGCUUAACAUCUGAUAGCUCUCCCAUAGGGAGACAACAAAUGUUAAACUGAUUUUUGGCAAGGGGAGGAAAGUUCGGGGCUUGCUCCACUUCUUCCGCGGGUUGGCCCGGUAUUGCAGUACCGCCGGGAUCGGCCCACAUUCAUUCUAUUAAACAAAAAUUGUG

..(((((((.((((....)))).)))....)))).................................(.((((((...)))))))................xxxxxxxxxxxx.(((((((.((..((((....)))))))))))))..(((((((((((((...........)))))).)))))))................(((.....))).

>7_14_u2_pre2_CM000061_5_7231897_7232087_Apis_mellifera_strain_DH4_linkage_group_8_whole_genome_shotgun_sequence

AUCGCUUCUCGGCCUGAUGGCUAAGAUCAAAGUGUAGUAUCUGUUCUUAUCAGCUUAAUAUCUGGUACACUCCCCACCGGGAGUCAGAAUAUUAGUCUGAUUUUUGGAACCGGGCGGAACCCCGGGGCUUGCUCCGCUUCUGCCGCGAGUCGGCUCGGCAUUGCAGUGCCGUCGAGAUCGGCUCAAUAAGUUACCCCAAAGAUUAAAGAAUU

..(((((((.((((....)))).)))....))))...........................(((....((((((....)))))))))............xxxxxxxxxxx..(((((((...(((((....))))).)))))))..(((((((((((((((....)))))...))).))))))).......................x....

>2_2_u2_pre2_GL341007_1_168927_169119_Nasonia_vitripennis_chromosome_4_unlocalized_genomic_scaffold_Chr4_random066_whole_genome_shotgun_sequence

AUCGCUUCUCGGCCUAUUGGCUAAGAUCAAAGUGUAGUAUCUGUUCUUAUCAGCUUGAUAUCUGAUACGCUCCUCAUUGAGGAGCCAGAAUAUCGAACUGAUUUUUGGAAUAUGGCGGAGUGUCUGGGGCUUGCUCCGACUCCGCCACGGGUCGACCUUGCAUUGCAGUACCGCAAGGAACGGCCCACACAAUUAAUUCAAACAAAAUCUUCUC

..(((((((.((((....)))).)))....))))....................((((.(((((....(((((((...)))))))))).)).))))....xxxxxxxxxxxx(((((((((...(((((....)))))))))))))).((((((.((((((...........))))))..))))))......................x.....

>7_1_u2_pre2_BX284601_5_12324944_12324754_Caenorhabditis_elegans_chromosome_I

AUCGCUUCUUCGGCUUAUUAGCUAAGAUCAAAGUGUAGUAUCUGUUCUUAUCGUAUUAACCUACGGUAUACACUCGAAUGAGUGUAAUAAAGGUUAUAUGAUUUUUGGAACCUAGGGAAGACUCGGGGCUUGCUCCGACUUCCCAAGGGUCGUCCUGGCGUUGCACUGCUGCCGGGCUCGGCCCAGUCCCCGAGGGGACAAAAUAAGCUUAC

..((((((((.((((....))))))))....))))......................(((((......(((((((....)))))))....))))).....xxxxxxxxxxxx..((((((..((((((....))))))))))))..((((((.((((((((......)).))))))..)))))).(((((....))))).......x.....

>7_5_u2_pre2_ABAV01032332_1_475_283_Nematostella_vectensis_strain_CH2_x_CH6_NEMVEscaffold_729_Cont32332_whole_genome_shotgun_sequence

AUCGCUUCUCGGCCUUUUGGCUAAGAUCAAGUGUAGUAUCUGUUCUUAUCAGCUUAAUAUCUGAUACGCUGCUCAUUGAGUAGCUCAUAUAUUAAACUGAUUUUUGGAAACUGGCUGUGGAAUAAGCGGCUUGCUGCGUCCCAGCCACGGGUUGUCUCGGUAUUGCACUACCUCCGAGUACGGCCCCCUUCCCUUUCGGGAAGACACAUUCAAG

..(((((((.((((....)))).)))...))))..................................(((((((...)))))))...............xxxxxxxxxxxx((((((.(((...(((.....)))...))))))))).((((((((((((.............))))).))))))).((((((....))))))...x.......

**U4**

>1_2_u4_pre_hs

AGCUUUGCGCAGUGGCAGUAUCGUAGCCAAUGAGGUCUAUCCGAGGCGCGAUUAUUGCUAAUUGAAAACUUUUCCCAAUACCCCGCCGUGACGACUUGCAAUAUAGUCGGCACUGGCAAUUUUUGACAGUCUCUACGGAGACUGAAUUUUU

.........(((((((((((((((.(((.....((.....))..))))))).))))))).))))....................((((((.(((((........))))).))).))).xxxxxxx.(((((((....))))))).......

>1_2_u4_pre_Dr

AGCUUUGCGCAGUGGCAGUAUCGUAGCCUAUGAGGUUUAUCCGAGGCGCGAUUAUUGCUAAUUGAAAACUUUACCCAAUACCCCGCCGUGACGACUUGAAAUAUAGUCGGCACUGGCAAUUUUUGACAGUCUUCUCGAAGACUGAAAUA

.........(((((((((((((((.((((..((......))..)))))))).))))))).))))....................((((((.(((((........))))).))).))).xxxxxxx.(((((((....))))))).....

>1_10_u4_pre_GG666612_1_8898552_8898412_Branc

AGCUUUGCGCAGAGGCGAUAUCAUAGCCAAUGAGGUCCAACCGAGGCGUGAUUAUUGCUAGUUGAAAACUUUUCCCAAUACCCCGCCUGGGGGACGUGAAAUACCGUCCACUAUGGCAAUUUUUGUGAGCCCCUACGGGGGCCCCCAU

.((.....))...(((((((((((.(((.....(((...)))..))))))).))))))).........................((((((.(((((........))))).))).))).xxxxxxx...(((((....)))))......

>1_3_u4_AABS01000396_1_28948_28808_Ciona_inte

AGCUUUGCGCAGUGGCGGUAUCGUAGCUGAUGAGGUUUAUCCGAGGCGCGAUUAUUGCUAGUUGAAAACUAUUACCAAUACCCCGCCCUGUCGACGUGAAAAACCGUCGACUGUGGCAAUUUCUGAUGGGUUUUCCGGAACAUUUCCU

.((.....))(.((((((((((((.(((.....((.....))..))))))).)))))))).)....................((((...(((((((........))))))).))))..xxxxxxx...........((((...)))).

>1_1_u4_pre_AAGJ05065406_1_6450_6590_Strongyl

AUCUUUGCGGAGAGGCAGUAUCGUAGCUAAUGAGGUUUAUCCGAGGCGCGAUUAUUGCUAGUUGAAAACUUAUCCCAAUACCCCGCUCUGACGACGUGAAAUACCGUGGGCUGUAGCAAUUUGUCCAGAUCUCCAAGGAGAUCUUACA

......((((...(((((((((((.(((.....((.....))..))))))).))))))).((((...........))))...))))..........((..(((((...)).)))..))xxxxxxx.(((((((....)))))))....

>1_2_u4_pre_AE014134_6_21215178_21215039_Dros

AUCUUUGCGCAGUGGCAAUACCGUAACCAAUGAAGUCCUCCUGAGGUGCGGUUAUUGCUAGUUGAAAACUUUAACCAAUACCCCGCCAUGGGGACGUGAAAUACCGUCCACUACGGCAAUUUUUGGAAGCCCGAGAGGGCUAAUUAAAUA

.........(((((((((((((((.(((................)))))))).))))))).)))....................(((.((((((((........))))).))).))).xxxxxxx..(((((....))))).........

>1_1_u4_pre_KB671676_1_581842_581982_Anophele

AGCUUUGCGCAGUGGCGAUAUCGUAACCAAUGAGGUACAACCGAGGUGCGAUUAUUGCUAGUUGAAAACUAAUACCAAUACCCCGCCUUGGGGACGUGAAAUACCGUCCGCUAUGGCAAUUUUUGGAAACCCCGAAAGGGGUCAUAAU

.........((((((((((((((((.((.....(((...)))..))))))).)))))))).)))....................(((.((((((((........))))).))).))).xxxxxxx...(((((....)))))......

>2_2_u4_pre_CM000054_5_13590219_13590079_Apis

AGCUUUGCGCAGUGGCGAUAUCGUAACCAAUGAGGUUCUACCGAGGUGCGAUUUUUGCUAGUUGAAAACUUUUACCAAUACCCCGCCAUGACGAUGUGAAAAAUCAUCGGCUACGGCAAUUUUUGGUAACCCCUACGGGGGUUUUUAA

.........(((((((((.((((((.((.....(((...)))..))))))))..)))))).)))....................(((.((.(((((........)))))..)).))).xxxxxxx..((((((....)))))).....

>1_2_u4_pre_Nvi

AUCUCUGCGCAGUGGCGAUAUCGUAACCAAUGAGGUUCUACCGAGGUGCGAUUAUUGCUAGUUGAAAACUUUUACCAAUACCCCGCCAAGACGAUGUGAAAUACCAUCGGCUACGGCAAUUUUUGACAGCCCUUACGAGGGUUAUAUU

.........((((((((((((((((.((.....(((...)))..))))))).)))))))).)))....................(((.((.(((((........))))).))..))).xxxxxxx..((((((....)))))).....

>1_5_u4_pre_Ce

AGCUUUGCGCUGGGGCGAUAACGUGACCAAUGAGGCUUUGCCGAGGUGCGUUUAUUGCUGGUUGAAAACUUUUCCCAAUUGCCCGCGAUGUCCCCUGAAACAUGGGUGGCAUACGCAAUUUUUGAAAGCCUCUAGGAGGCAGAAAAACAUCUUC

(((.....)))..(((((((((((.(((.....(((...)))..)))))).)))))))).........................(((((((((((........))).))))).))).xxxxxxx...(((((...)))))..............

>1_7_u4_pre_ABAV01042195_1_334_472_Nematostel

AGCUUUGCGCAGUGGCAUUACCGUAGCUGAUGAGGUCCAUCCGACGCGCGGUUAUUGCUGAUUGAAAACUUUUCCCAAUACCCCGCGAGACGACUUGAAACAUAGUCGGCUUUGCAAUUUUUGUGGGCCCUAUUUAGGGCCACCAU

.........((((((((..(((((.((.((((.....))))....)))))))...)))).))))....................(((((.(((((........)))))..))))).xxxxxxx..((((((....)))))).....

**U5**

>4_20_u5_pre5_CM000059_5_9966625_9966505_Apis_mellifera_strain_DH4_linkage_group_3_whole_genome_shotgun_sequence

GUACUCUGGUUUCCCUUCAAAUCACGCAUAAAUCUUUCGCCUUUUACUAAAGAUUUCCGUGGAGGGGAACAAUUGAUGAGUCUAUAGACUAAUUUUUUGUAUACCCGGCGCAAGCUGGGUCAUUAAUAAAAUAAAACAUAAUUAGUAAUUUAUUUUUAUAUUUGUGUAU

..((((..(((((((((.....((((...((((((((.(.......).)))))))).))))))))))))).......))))..........xxxxxxxxxxx(((((((....)))))))...............((((((...((((.......))))..))))))..

>11_10_u5_pre5_CM002889_2_43123291_43123176_Danio_rerio_chromosome_5_GRCz11_reference_primary_assembly

CAGCUCGAGUUUCUCUUCAAACACGCACAAAUCUUUCGCCUUUUACUAAAGAUUUCCGUGGGGAGGAACUAUUGUGAGUUAUGUUUAUUUUUGGGUGCUCUGCUAUCUGCAGAGCUGCAUUAAGAUGUUGAAUUGCAGAACGAGAUGUGGAAGAGUGUUUGUGU

..((((((((((((((((....(((...((((((((.(.......).)))))))).))))))))))))))....))))).......xxxxxxxxxx(((((((.....)))))))...................(((((((.............)).)))))..

>5_8_u5_pre5_GG666485_1_1370472_1370594_Branchiostoma_floridae_genomic_scaffold_BRAFLscaffold_37_whole_genome_shotgun_sequence

UCACUCUGGUUUCCCUUCAAUCCACCACGCACAAAUCUUUCGCCUUUUACUAAAGAUUUCCGUGGAGGGGAACAAUCAAUGAGUCUAUAAACAAUUUUUCUCUGCCCUGCCUUGUGCAGGGCUCCUAAUAAACCAAAGAAGUCCACACCCAACACACCUUUGCUAAGCCUA

..((((..((((((((........(((((...((((((((.(.......).)))))))).))))))))))))).......)))).........xxxxxxxxx.(((((((.....)))))))...........(((((..((...........))..))))).........

>2_9_u5_pre5_AABS01000196_1_104341_104227_Ciona_intestinalis_ciona0196_whole_genome_shotgun_sequence

CCACUCUGGCUUCUCUUCAGCGCAUGAUUCUUUCGCCUUUUACUAAAGAAUUCCGUGGAGGGGAGCAUUUCAAUGAGUCUUUAACAAUUUUUCUUUACCCGAUUUCGGUCGGGCUCCUAUUUUUAACCAUUUUUUUAUAUGUAUCGAUUACGAGCUGUAAAUG

..((((..(((((((((((.((...((((((((.(.......).)))))))).)))))))))))))........))))........xxxxxxxxx..((((((....))))))....................((((((.((.(((....)))))))))))..

>7_17_u5_pre5_AAGJ05103420_1_12808_12925_Strongylocentrotus_purpuratus_Contig103420_fixed_whole_genome_shotgun_sequence

AUGGGUGGUUUUUCUGCAAUACGCAUAAAUCUUUCGCCUUUUACUAAAGAUUUCCGUGCAGAGGAACACAUUGAUGAGUCUAUAAAGAUUUUUCUCUGGCUUAAAAUUAUUUGAUGCAAAGGAAUUCAAUCGAUCGAUGAACAGGCUCUGUCCUACCAACAUCGAU

((.((((((((((((((...(((...((((((((.(.......).)))))))).))))))))))))).)))).))............xxxxxxxxx..(((((((....))))).))................((((((...(((......))).....)))))).

>1_2_u5_pre5_AE014134_6_19812075_19811954_Drosophila_melanogaster_chromosome_2L

UUACUCUGGUUUCUCUUCAAUUGUCGAAUAAAUCUUUCGCCUUUUACUAAAGAUUUCCGUGGAGAGGAACACUCUAAUGAGUCUAAACUCAAUUUUUGUAUGACCUGGCUAAAUAUUUAGUUGGGCCAAUGAAUAAUAAAUAAAAAUGAACGAAAUCCGUUCGAUGAAGG

....(((.(((((((((((.....((...((((((((.(.......).)))))))).)))))))))))))........((((....))))xxxxxxxxxxxx.(((((((((....)))))))))......................(((((.....))))).....)))

>1_5_u5_pre5_CM000359_1_15809445_15809564_Anopheles_gambiae_str_PEST_chromosome_3R_whole_genome_shotgun_sequence

GCACUCUGAUCUCUCUUCAACUGUCGAAUAAAUCUUUCGCCUUUUACUAAAGAUUUCCGUGGAGAGGGAUACUCUAAUGAGUCUAUAGUGAAUUUUUGUCCGUCUCGAUUCCGUAAGGAGUCGAGCCCUAAACUUCAAUACAAAAAACUUAUAGCAUAAAUCUGUGGA

.(((...((((((((((((.....((...((((((((.(.......).)))))))).))))))))))))).)...................xxxxxxx.....(((((((((....)))))))))......................................)))..

>4_16_u5_pre5_CM000677_2_65296051_65296166_Homo_sapiens_chromosome_15_GRCh38_reference_primary_assembly

AUACUCUGGUUUCUCUUCAGAUCGCAUAAAUCUUUCGCCUUUUACUAAAGAUUUCCGUGGAGAGGAACAACUCUGAGUCUUAACCCAAUUUUUUGAGGCCUUGCUUUGGCAAGGCUAUAUGUGGUAAUCCAACAAUAGAAAUUAUUUUUAAGUUUGUGUGUUCC

..((((..(((((((((((...((...((((((((.(.......).)))))))).)))))))))))))......)))).........xxxxxxxxxxxxx((((....))))..................((((.((.(((((.......))))).))))))..

>2_5_u5_pre5_CM000918_2_9172767_9172648_Nasonia_vitripennis_chromosome_4_whole_genome_shotgun_sequence

UUACUCUGGUUUCCCUUCAUUACACGCAUAAAUCUUUCGCCUUUUACUAAAGAUUUCCGUGGAGGGGAACACUUGAUGAGUCUAUAAAAAAUUUUUUGUGUGCCCGACGAAAGUUGGGCCGUUAUAUAAUUUAAAAAUAAUUCGACCUAGUGAUCGAUCACGUAAUCG

..((((..((((((((((....((((...((((((((.(.......).)))))))).)))))))))))).)).....)))).........xxxxxxxx...(((((((....))))))).......................(((....((((....))))....)))

>2_8_u5_pre5_BX284604_4_9444641_9444762_Caenorhabditis_elegans_chromosome_IV

UAACUCUGGUUCCUCUGCAUUUAACCGUGAAAAUCUUUCGCCUUUUACUAAAGAUUUCCGUGCAAAGGAGCAUACAUUGAGUAUUAUAUACAAUUUUUGGAGUCCCCUUGAGAAAGCGGGACAAAAGUUUGGAGCAGUAUUACACGAUAUGAAUGCAAAUUCAUUUAUUG

..((((..((((((.((((......((.(.((((((((.(.......).))))))))))))))).)))))).......))))..........xxxxxxx..((((((((....))).)))))......................((((((((((....)))))..)))))

>1_6_u5_pre5_ABAV01015157_1_376_255_Nematostella_vectensis_strain_CH2_x_CH6_NEMVEscaffold_140_Cont15157_whole_genome_shotgun_sequence

GCACUCUGGUUUUCCUCCAUAUCGAGUAAAUCUUUCGCCUUUUACAAAAGAUUUCCGUCGAGGAGAGCACUGAAAUGAGUAUAUCACUCAAUUUUUGAUUUGCCCUGCAUUUUUGCGGGGCUUACACACUAAAUGAAAAAUUGCGGCCCAAAUGGUUCAACUGAAUUUUG

..((((..(((((((((.....((.(.((((((((.(.......).)))))))))))..)))))))))........))))..........xxxxxxxx...((((((((....))))))))...........................(((...((((....)))).)))
