# Supplementary material for: The SMN complex drives structural changes in human snRNAs to enable snRNP assembly
Source: Nat Commun. 2023 Oct 18;14:6580. doi: 10.1038/s41467-023-42324-0 (PMC10584915; doi:10.1038/s41467-023-42324-0)
Supplement: Supplementary file 7 — Supplementary Software 1 [file 41467_2023_42324_MOESM7_ESM.zip › Supplementary file-code/README.docx]

This is the README file describing the software code for the computational part of the following work:

The SMN complex drives structural changes in snRNAs to enable snRNP assembly

by

Josef Pánek^1,# ,*^, Adriana Roithová^2,#,§^, Nenad Radivojević^2^, Michal Sýkora^2^, Archana Prusty^3^, Nicholas Huston^4^, Han Wan^4^, Anna Marie Pyle^5,6,7^, Utz Fischer^3^, David Staněk^2,*^

General:

The theoretical background for the provided code is explained in the Computational procedures section in Methods. Contacting the authors is strongly recommended before the use of the presented code, as the code is not a classical input –> code –> output software, but rather computational analytical tools that are used with several manual steps.

The code uses both Matlab and Linux bash programing languages. Running the Matlab scripts (*.m) requires both a Linux operating system and the Matlab computational environment with the Bioinformatics and Statistics toolboxes. The scripts contain a substantial portion of the Linux bash code. For the presented work, Matlab v. 2019a and CentOS 7 were used, nevertheless, the code is version-independent. No installation is required for the scripts. No software repository was used to provide the code as it is a single-use, single–purpose and single-version software, and there will be no future development. No demo data is provided as the analysis includes manual steps. The results of the computation using the provided scripts is not self-explanatory and should be interpreted visually.

The code scripts:

IDENTIFICATION OF BEST REPRESENTATIVE SUBOPTIMAL STRUCTURE

1. A manual download of snRNA sequences from Rfam and/or NCBI.

2. get_u_sqs_from_Rfam.m. Extracts sequences of snRNAs from Rfam files.

3. get_sqs_from_NCBI.m. Extracts sequences of snRNAs from NCBI files.

4. motifs.m. Contains Sm motifs for individual snRNAs.

5. filter_sqs_by_motif.m. Filter out snRNAs with sequences without Sm motif, and in the case of U1 snRNA without U1-70K binding motif.

6. get_fl_sqs_locally_better.m. Gets extra sequences for snRNAs.

7. add_info.m. U1 snRNA only. Adds a folding constraint for U1-70K binding motif.

8. best_strs.m. Computes best suboptimal structure for individual snRNAs. find_tm.m. Finds a template among best suboptimal structures that is best matching among suboptimal structures of all species.

Optional:

call_u.m. Runs the whole workflow for the identification of best representative suboptimal structure.

THE STRUCTURE REARRANGEMENT

1. Manually set folding constraints for folding intermediates and final structures (as it is shown in Supplementary files S1 and S2).

2. Run constrained suboptimal secondary structure prediction (RNAsubopt –C) using the Linux shell command line for the snRNA sequences with folding constraints in Supplementary files S1 and S2.

3. f_reformat_RNAsubopt.m. Reformates the RNAsubopt output for further use.

4. f_test_sopts_various_sqs.m. Computes the best representative suboptimal structures for folding intermediates and final structures.
