# Supplementary material for: The SMN complex drives structural changes in human snRNAs to enable snRNP assembly
Source: Nat Commun. 2023 Oct 18;14:6580. doi: 10.1038/s41467-023-42324-0 (PMC10584915; doi:10.1038/s41467-023-42324-0)
Supplement: Supplementary file 8 — Reporting Summary [file 41467_2023_42324_MOESM8_ESM.pdf]

Corresponding author(s): David Stanek, Josef Panek

Last updated by author(s): Sep 14, 2023

Reporting Summary

Nature Portfolio wishes to improve the reproducibility of the work that we publish. This form provides structure for consistency and transparency in reporting. For further information on Nature Portfolio policies, see our Editorial Policies and the Editorial Policy Checklist.

Statistics

For all statistical analyses, confirm that the following items are present in the figure legend, table legend, main text, or Methods section.

n/a	Confirmed	
☐	☒	The exact sample size (*n*) for each experimental group/condition, given as a discrete number and unit of measurement
☐	☒	A statement on whether measurements were taken from distinct samples or whether the same sample was measured repeatedly
☐	☒	The statistical test(s) used AND whether they are one- or two-sided<br>*Only common tests should be described solely by name; describe more complex techniques in the Methods section.*
☒	☐	A description of all covariates tested
☒	☐	A description of any assumptions or corrections, such as tests of normality and adjustment for multiple comparisons
☐	☒	A full description of the statistical parameters including central tendency (e.g. means) or other basic estimates (e.g. regression coefficient) AND variation (e.g. standard deviation) or associated estimates of uncertainty (e.g. confidence intervals)
☐	☒	For null hypothesis testing, the test statistic (e.g. *F*, *t*, *r*) with confidence intervals, effect sizes, degrees of freedom and *P* value noted<br>*Give P values as exact values whenever suitable.*
☒	☐	For Bayesian analysis, information on the choice of priors and Markov chain Monte Carlo settings
☒	☐	For hierarchical and complex designs, identification of the appropriate level for tests and full reporting of outcomes
☒	☐	Estimates of effect sizes (e.g. Cohen's *d*, Pearson's *r*), indicating how they were calculated

Our web collection on statistics for biologists contains articles on many of the points above.

Software and code

Policy information about availability of computer code

Data collection	SHAPE libraries were quantified using Qubit (Invitrogen) and BioAnalyzer (Agilent).
Data analysis	Microscopy images were deconvolved using the SoftWoRx software v7.0.0 (GE Healthcare). Fluorescent intensities in the micrographs were analyzed using the Fiji software 1.48i.<br><br>All SHAPE data was analyzed using the ShapeMapper 2 Differences in SHAPE reactivities were analyzed the DeltaSHAPE automated analysis tool and default settings.<br><br>Identification of the suboptimal pre-snRNA structures , we used a code based on Matlab and Linux bash programing languages. Running the Matlab scripts requires both a Linux operating system and the Matlab computational environment with the Bioinformatics and Statistics toolboxes. The scripts contain a substantial portion of the Linux bash code. For the presented work, Matlab v. 2019a and CentOS 7 were used. The detail description is provided in the Supplementary Software file.

For manuscripts utilizing custom algorithms or software that are central to the research but not yet described in published literature, software must be made available to editors and reviewers. We strongly encourage code deposition in a community repository (e.g. GitHub). See the Nature Portfolio guidelines for submitting code & software for further information.

Data

Policy information about availability of data

All manuscripts must include a data availability statement. This statement should provide the following information, where applicable:

- Accession codes, unique identifiers, or web links for publicly available datasets
- A description of any restrictions on data availability
- For clinical datasets or third party data, please ensure that the statement adheres to our policy

All data supporting the findings of this study are available within the paper and its Supplementary Information. The RNA-seq data used for SHAPE-MaP are accessible at ArrayExpress (www.ebi.ac.uk/biostudies/arrayexpress) using accession # E-MTAB-13248. All accession numbers and links for pre-snRNA sequences used in this study are provide in Supplementary Data 1.

Research involving human participants, their data, or biological material

Policy information about studies with human participants or human data. See also policy information about sex, gender (identity/presentation), and sexual orientation and race, ethnicity and racism.

Reporting on sex and gender	n/a
Reporting on race, ethnicity, or other socially relevant groupings	n/a
Population characteristics	n/a
Recruitment	n/a
Ethics oversight	n/a

Note that full information on the approval of the study protocol must also be provided in the manuscript.

Field-specific reporting

Please select the one below that is the best fit for your research. If you are not sure, read the appropriate sections before making your selection.

☒ Life sciences ☐ Behavioural & social sciences ☐ Ecological, evolutionary & environmental sciences

For a reference copy of the document with all sections, see nature.com/documents/nr-reporting-summary-flat.pdf

Life sciences study design

All studies must disclose on these points even when the disclosure is negative.

Sample size	No statistical method was applied to predetermine the sample size. The sample size follows common standards in the field employing three or more biological replicates for experiments. This number is based on extensive laboratory experience and literature in the field. The sample size is always included in the figures or figure legend.<br><br>For microinjection experiments, we analyzed between tens of injected cells. The number is always indicated in the graphs. These sample sizes provided us with enough data points to adequately test the statistical significance of the observed changes in Cajal body accumulation (Figs. 3, 5, 6).<br><br>Four independent experiments were performed to analyze molecular beacon relaxation by the SMN complex. One experiment was excluded due to unsubstantiated low value of annealed probed at the level of background.
Data exclusions	One experiment with molecular beacon was excluded due to unsubstantiated low value of annealed probed at the level of background.
Replication	Number of replication for each experiment is stated in the figure legend.
Randomization	Cells were plated into different well under identical conditions and then randomly allocated for control or siRNA treatment and microinjection.
Blinding	Neither of the experiment presented in the manuscript has been performed as double-blinded. The only relevant experiment where blinding would would be relevant is analysis of microinjected cells. However, there are only a few cells injected per plate, which contains millions of cells making it extremely difficult for blinded person to identify the injected ones. To overcome this issue, results of microinjection experiments were always double-controlled by a supervisor (D.S.).

Reporting for specific materials, systems and methods

We require information from authors about some types of materials, experimental systems and methods used in many studies. Here, indicate whether each material, system or method listed is relevant to your study. If you are not sure if a list item applies to your research, read the appropriate section before selecting a response.

Materials & experimental systems

n/a	Involved in the study
☐	☒ Antibodies
☐	☒ Eukaryotic cell lines
☒	☐ Palaeontology and archaeology
☒	☐ Animals and other organisms
☒	☐ Clinical data
☒	☐ Dual use research of concern
☒	☐ Plants

Methods

n/a	Involved in the study
☒	☐ ChIP-seq
☒	☐ Flow cytometry
☒	☐ MRI-based neuroimaging

Antibodies

Antibodies used

Primary antibodies: Anti-Gemin3 (mouse monoclonal, clone 12H12, Abcam, catalog# ab10305, lot GR11126-13, dilution 1:400), anti-Gemin4 (mouse monoclonal, clone 3E1, Sigma, catalog# WH0050628M1-100UG, lot 07334-3E1, dilution 1:500) and anti-Gemin5 (mouse monoclonal, clone 10G11, SantaCruz Biotechnology, catalog# sc136200, lot C1413, dilution 1:500) were used for western blotting. Mouse monoclonal anti-SMN antibody (clone 7B10 94, ImmunoGlobe, catalog# 0176-01) used for the SMN complex purification was prepared from original hybridoma cell line in the laboratory of Utz Fischer by Archana Prusty (Department of Biochemistry, Theodor Boveri Institute, University of Würzburg). This hybridoma cell line established and validated by by Utz Fischer team (Meister et al. 2000, Hum. Mol. Gen.). Mouse monoclonal anti-coilin (5P10) antibody was kindly provided by M. Carmo-Fonseca (Institute of Molecular Medicine, Lisboa). For immunoprecipitation, we used anti-Sm Y12 antibody produced from a hybridoma cell line (a gift from Karla Neugebauer, Yale University, New Haven, USA) at the Antibody Facility (Institute of Molecular Genetics of the Czech Academy of Sciences).
Secondary antibodies: Anti-mouse antibodies conjugated with Alexa-647 (Thermo Fisher Scientific, Cat No. A21236) were used for immunofluorescence and peroxidase-conjugated anti-mouse IgG (Jackson ImmunoResearch Laboratories, Cat No. 115-035-003) and peroxidase-conjugated anti-rabbit IgG (Jackson ImmunoResearch Laboratories, Cat No. 111-035-003) for western blotting.

Validation

All the antibodies were validated by a provider by Western blotting and correct target size was checked at western blot and by RNAi. The mouse monoclonal anti-Sm (Y12) was produced from a hybridoma clone in our Antibody producing facility and validated by western blotting in cells knocked down for SmB or in cells where the Y12 antibody epitope in SmB was removed. Validation data can be provided upon request. Anti-coilin 5P10 specificity was validated in coilin knockout cells (Basello et al. 2022, J. Cell Sci.). Specificity of anti-Gemin3, Gemin4 and Gemin5 antibodies were validated in the current manuscript using siRNA or Gemin3 depletion (Figs. S17 and S18). The monoclonal anti-SMN antibody was validated in Meister et al. 2000, Hum. Mol. Gen., Fig. 1). Y12 antibody, which primarily recognizes SmB/B' proteins was validated in Roithova et al. 2018, Nuc. Acids. Res. by siRNA downregulation of SmB/B' proteins (Fig. S3).

Eukaryotic cell lines

Policy information about cell lines and Sex and Gender in Research

Cell line source(s)

HeLa cell line (female origin) used in the study were received from prof. Karla Neugebauer and we have all reasons to believe that they originated from ATCC® HeLa-CCL-2 strain. They were further characterized in the publication Hebert et al. Dev. Cell 2002, https://doi.org/10.1016/S1534-5807(02)00222-8 as HeLa KN. We have been using this HeLa strain in many previous publications (e.g. Klimesova et al. 2021, Nature Comm., Novotny et al. 2015, Cell Report, Roithova et al. 2020 Nuc. Acids Res.). This HeLa strain was further used to establish the Gemin3-degron cell line (HeLa-DDX20-EGFP-FKBP12F36V), which is characterized in this manuscript. We also utilized HeLa S3 cell (ATCC® CCL-2.2™).

Authentication

None of the cell lines used was specifically validated.

Mycoplasma contamination

All cell lines were regularly every 2-3 months tested for mycoplasma contamination in the Antibody production facility at the Institute of Molecular Genetics using MycoAlert PLUS Mycoplasma Detection Kit. All cells used in the study were mycoplasma-free.

Commonly misidentified lines
(See ICLAC register)

HeLa cells are not listed in ICLAC register of commonly misidentified lines.
